# microRNA-21 Governs TORC1 Activation in Renal Cancer Cell Proliferation and Invasion

**DOI:** 10.1371/journal.pone.0037366

**Published:** 2012-06-04

**Authors:** Nirmalya Dey, Falguni Das, Nandini Ghosh-Choudhury, Chandi Charan Mandal, Dipen J. Parekh, Karen Block, Balakuntalam S. Kasinath, Hanna E. Abboud, Goutam Ghosh Choudhury

**Affiliations:** 1 Department of Medicine, University of Texas Health Science Center at San Antonio, San Antonio, Texas, United States of America; 2 Veterans Administration Research, South Texas Veterans Health Care System, San Antonio, Texas, United States of America; 3 Geriatric Research, Education and Clinical Center, South Texas Veterans Health Care System, San Antonio, Texas, United States of America; 4 Department of Pathology, University of Texas Health Science Center at San Antonio, San Antonio, Texas, United States of America; 5 Department of Urology, University of Texas Health Science Center at San Antonio, San Antonio, Texas, United States of America; Children's Hospital Boston & Harvard Medical School, United States of America

## Abstract

Metastatic renal cancer manifests multiple signatures of gene expression. Deviation in expression of mature miRNAs has been linked to human cancers. Importance of miR-21 in renal cell carcinomas is proposed from profiling studies using tumor tissue samples. However, the role of miR-21 function in causing renal cancer cell proliferation and invasion has not yet been shown. Using cultured renal carcinoma cells, we demonstrate enhanced expression of mature miR-21 along with pre-and pri-miR-21 by increased transcription compared to normal proximal tubular epithelial cells. Overexpression of miR-21 Sponge to quench endogenous miR-21 levels inhibited proliferation, migration and invasion of renal cancer cells. In the absence of mutation in the PTEN tumor suppressor gene, PTEN protein levels are frequently downregulated in renal cancer. We show that miR-21 targets PTEN mRNA 3′untranslated region to decrease PTEN protein expression and augments Akt phosphorylation in renal cancer cells. Downregulation of PTEN as well as overexpression of constitutively active Akt kinase prevented miR-21 Sponge-induced inhibition of renal cancer cell proliferation and migration. Moreover, we show that miR-21 Sponge inhibited the inactivating phosphorylation of the tumor suppressor protein tuberin and attenuated TORC1 activation. Finally, we demonstrate that expression of constitutively active TORC1 attenuated miR-21 Sponge-mediated suppression of proliferation and migration of renal cancer cells. Our results uncover a layer of post-transcriptional regulation of PTEN by transcriptional activation of miR-21 to force the canonical oncogenic Akt/TORC1 signaling conduit to drive renal cancer cell proliferation and invasion.

## Introduction

Renal cell carcinoma represents the most common kidney malignancy; about 70,000 new cases have been reported in the year 2011 (www.cancer.gov). Among the five subtypes, clear cell renal carcinoma (RCC) accounts for about 70% of the cases [1]. About 30% of patients with RCC develop invasive disease commonly metastasizing to bone, lung, brain and liver [2], [3]. Loss of VHL (von Hippel-Lindau) protein expression due to germline mutation, biallellic somatic mutation or hypermethylation of its gene locus poses a high risk for clear cell renal carcinoma, hemangiomas and pheochromocytomas [4], [5]. Defective VHL expression causes stabilization of Hifα transcription factors, which contribute to the increased expression of vascular endothelial growth factor (VEGF) to maintain vascular nature of the tumor. Also, Hifα regulates anaerobic respiration often found in RCC [5]. Hifα-independent function of VHL has been reported in driving kidney carcinoma, including regulation of senescence [5], [6]. Furthermore, VHL positive kidney tumors utilize alternative mechanisms to increase Hifα transcription factors for VEGF expression, and, Hifα-independent growth factor receptor upregulation [5], [7].

miRNAs are short noncoding oligonucleotides with imperfect complementarity predominantly to the 3′untranslated region (UTR) of target mRNAs [8], [9], [10]. Nearly 1000 miRNAs in humans regulate the expression of one third of the total protein coding transcriptome at the posttranscriptional and translational level [9]. miRNAs predominantly act by inhibiting mRNA translation although mRNA degradation and mRNA cleavage may also contribute to downregulation of protein levels. Inappropriate expression of miRNAs have been linked to oncogenesis [10], [11]. miRNAs are coded by the intronic and intergenic as well as exon sequences in the genome [12]. They are synthesized predominantly by the RNA polymerase II-dependent transcription to produce pri-miRNA hairpin, which binds Drosha/DGCR8 complex. The double stranded RNA-binding protein DGCR8 recognizes the proximal bases (∼ 10 bp) of the pri-miRNA stem followed by its cleavage by the RNase III enzyme Drosha to release the pre-miRNA short hairpin [13]. Exportin-5 and its partner Ran-GTP induce nuclear export of the pre-miR to the cytoplasm where it is processed by the dicer RNase III/TRBP to yield ∼22 nucleotide small RNA duplex. The guide strand then is incorporated into effector Argonaute complex to form RISC (RNA-induced silencing complex) and to bind with imperfect complementarity to the mRNA for translational repression [12].

Recent reports established a firm role of specific miRNA signature in renal tumorigenesis. Profiling experiments showed that more miRNAs are downregulated in RCC than upregulated [14], [15], [16], [17]. For example, in an initial screen of 470 miRNAs, only six miRNAs were found to be upregulated in RCC while 15 were downregulated [16]. In another study, only 2 miRNAs were increased in RCC including miR-21 whereas the expression of 17 miRNAs was decreased [17]. Similarly, a more recent report showed increased expression of miR-21 among 9 miRNAs while the expression of 26 miRNAs was suppressed [14]. Recently, an extensive study using a large number of cancer samples from 31 different solid tumors described a significant increase in miR-21 suggesting its function in oncogenesis [18]. However, its functional role in many cancers including renal carcinoma has not been elucidated. In the present study, we find increased expression of mature, pre- and pri-miR-21 in renal cancer cells as compared to normal proximal tubular epithelial cells. This increase in miR-21 was associated with decreased PTEN levels. Neutralization of miR-21 prevented proliferation, migration and invasion of these cells concomitant with increased PTEN and reduced tuberin phosphorylation and mTORC1 activation.

## Results

### Increased Expression of miR-21 in Renal Cell Carcinoma

**Figure 1:**
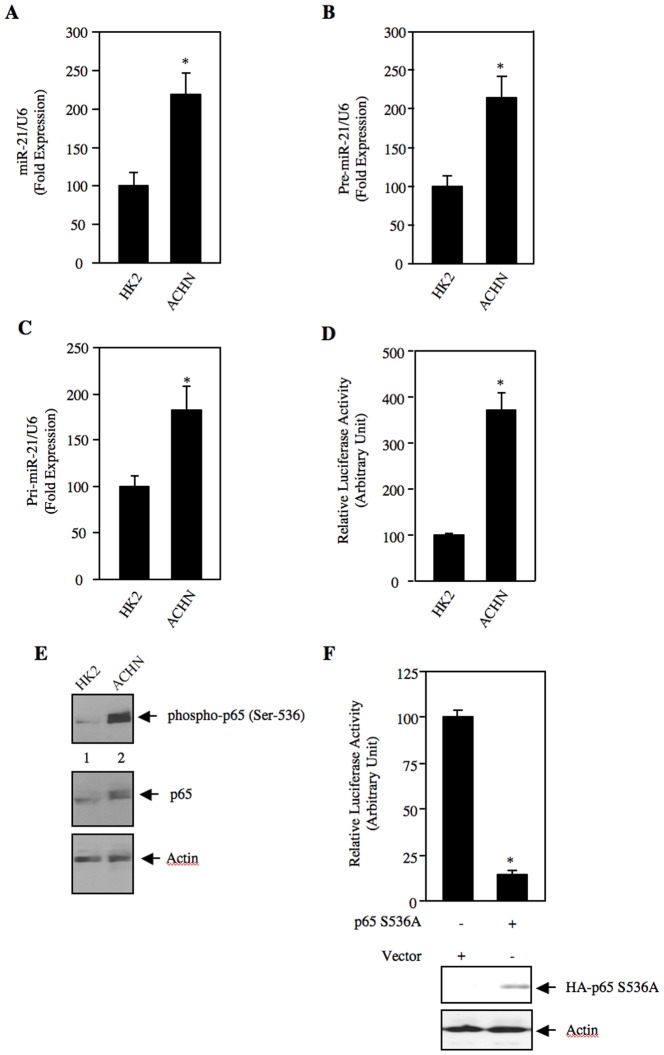
Expression of miR-21 in renal cancer cells. (A – C) Total RNAs from HK2 proximal tubular epithelial cells and ACHN renal cancer cells were used to detect mature miR-21 (panel A), pre-miR-21 (panel B) and pri-miR-21 (panel C) by real time qRT-PCR as described in the Materials and Methods. Mean ± SE of 4 measurements is shown. In panels A and B, *p  = 0.004 vs HK2 cells. In panel C, *p  = 0.02 vs HK2 cells. (D) HK2 and ACHN cells were transfected with miR-21-Luc reporter along with Renilla null plasmid. The cell lysates were used to determine luciferase activity as described in the Materials and Methods. Mean ± SE of 6 measurements is shown. *p  = 0.001 vs HK2 cells. (E) Increased phosphorylation of p65 NFkB in renal cancer cells. Lysates of HK2 and ACHN cells were immunoblotted with phospho-p65 (Ser-536), p65 and actin antibodies. (F) Mutant p65 S536A inhibits miR-21 transcription. ACHN renal cancer cells were cotransfected with miR-21-Luc and p65 S536A expression vector. The luciferase activity was determined in the cell lysates. Mean ± SE of quadruplicate measurements is shown. *p  = 0.02 vs vector. Bottom panels show expression of the HA-tagged p65 S536A and actin.

miRNA expression profiling using tumor samples from patients with renal carcinoma has been reported. In three studies, the expression of miR-21 was found to be increased [14], [17], [19]. However, in another study in 16 clear cell renal carcinoma and 4 chromophobe renal carcinoma samples, miR-21 was not detected [16]. We used a panel of tumor tissues from clear cell renal carcinomas to detect the expression of mature miR-21. Real time qRT-PCR revealed markedly increased expression of mature miR-21 in all grade 2 (3 out of 3) and grade 3 (3 out of 3) renal cell carcinoma samples (Fig. S1A and S1B). Next, we investigated the expression of miR-21 in ACHN renal carcinoma cells. We found significantly increased expression of mature miR-21 in ACHN cells as compared to the HK2 normal proximal tubular epithelial cells (Fig. 1A). Similar results were obtained in another renal cancer cell line Caki-2 (Fig. S2). Recent reports showed that miR-21 is regulated at the level of maturation [20]. Therefore, we examined the levels of pre-miR-21. Real time qRT-PCR showed marked expression of pre-miR-21 in ACHN cells compared to HK2 cells (Fig. 1B). Similarly, increased expression of pri-miR-21 was also detected in the ACHN cells (Fig. 1C). This increase in pri-miR suggested a transcriptional mechanism of miR-21 expression. To directly examine the transcriptional regulation of miR-21 expression, we used a miR-21 promoter-driven firefly luciferase reporter construct (miR-21-Luc). Transient transfection assays in ACHN cells revealed significantly increased transcription of the reporter gene (Fig. 1D). These results indicate that enhanced levels of mature miR-21 in renal carcinoma cells may result from the increased transcription of miR-21 gene to produce pri-mir-21.

Transcription of miR-21 gene has recently been shown to be regulated by NFκB [21]. NFκB is upregulated in renal cancer [22], [23]. The transcriptional activity of the p65 subunit of NFκB is dependent upon its phosphorylation at Ser-536 [24]. Therefore, we examined phosphorylation of p65. As shown in Fig. 1E, phosphorylation of p65 at Ser-536 was significantly increased in the ACHN renal cancer cells compared to the HK2 cells. Furthermore, enhanced p65 levels were also observed (Fig. 1E, middle panel). Next, we tested the involvement of NFκB in transcription of miR-21 in renal cancer cells. miR-21-Luc reporter was cotransfected with either the S536A mutant of p65 or with a vector plasmid. Expression of p65 S536A significantly inhibited the reporter activity in ACHN cells. These data indicate that upregulation of miR-21 may partly be due to increased NFκB activity in renal cancer cells.

### miR-21 Regulates Proliferation and Invasion of Renal Cancer Cells

**Figure 2:**
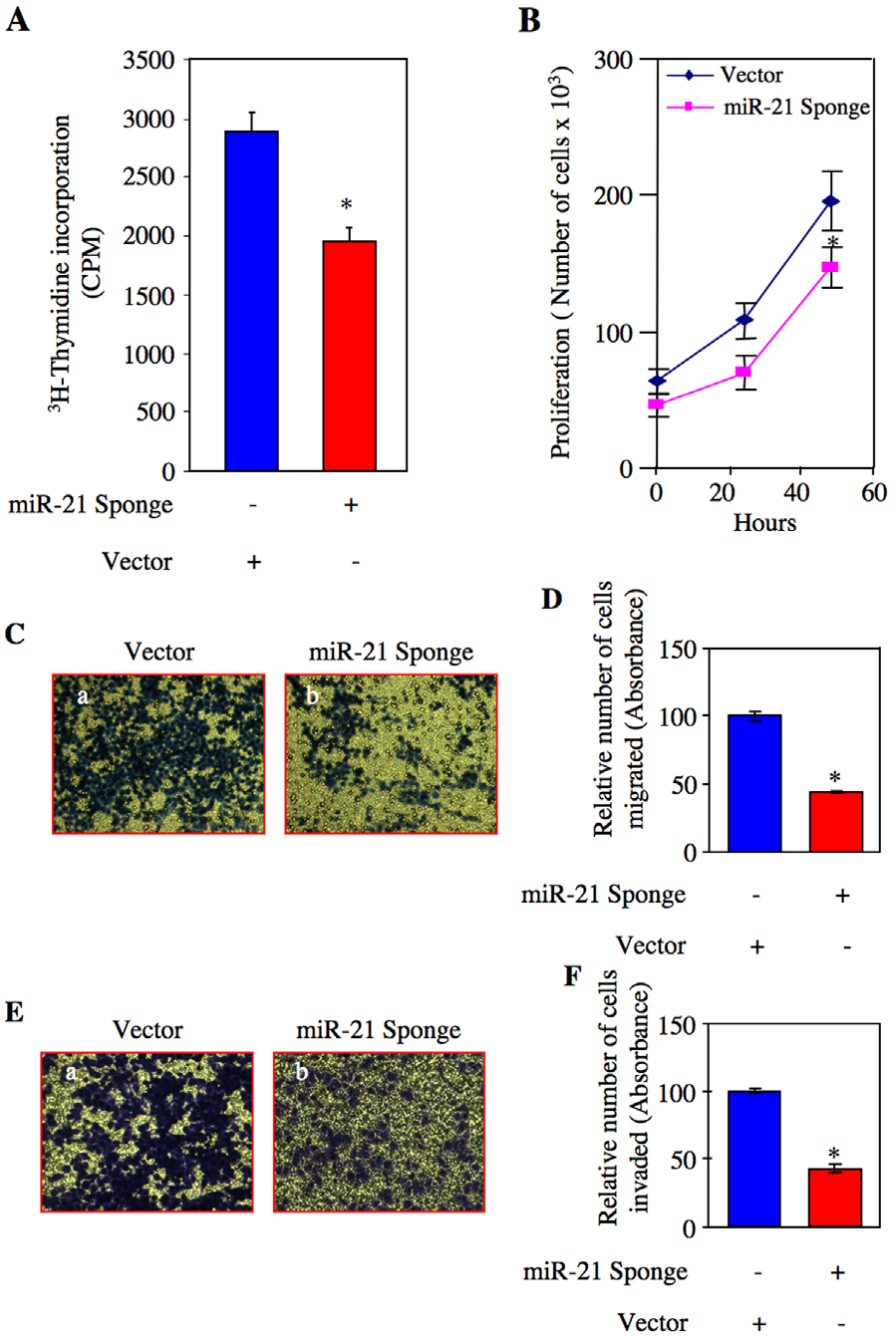
Effect of miR-21 Sponge on proliferation, migration and invasion of renal cancer cells. ACHN cells were transfected with miR-21 Sponge or vector plasmids. (A) Serum-starved cells were incubated with ^3^H-thymidine and its incorporation into DNA was determined as described in the Materials and Methods [115]. Mean ± SE of 12 measurements is shown. *p  = 0.004 vs vector alone. (B) The cells were counted at indicated time periods as described in the Materials and Methods. Diamond and square symbols represent vector and miR-21 Sponge-transfected cells, respectively. Means ± SE of triplicate measurements are shown. *p<0.05 vs vector alone. (C) Serum-starved cells were seeded on to membrane in a trans-well chamber. Migration assay was performed and the migrated cells at the opposite side of the membrane were stained as described in the Materials and Methods [111]. (D) The stains in the migrated cells in panel C were eluted as described in the Materials and Methods [111]. Mean ± SE of triplicate measurements is shown. *p<0.0001 vs vector-transfected cells. (E) Transfected serum-starved cells were seeded on to collagen-embedded membrane in a trans-well chamber. Invasion assay was performed and the invaded cells at the opposite side of the membrane were stained as described in the Materials and Methods [111]. (F) The stains in the invaded cells in panel E were eluted as described in the Materials and Methods [111]. Mean ± SE of triplicate measurements is shown. *p<0.001 vs vector-transfected cells.

miR-21 is considered to be an onco-miR in many cancers. However, its role in renal cell carcinoma has not been explored. Therefore, we tested the involvement of miR-21 in mitogenesis of ACHN cells, using a plasmid vector expressing seven copies of the bulged miR-21 binding site placed in the 3′ end of CMV promoter-driven GFP mRNA (Fig. S3) [25]. Expression of this construct serves as a “Sponge” that quenches the levels of endogenous miR-21 [25], [26]. ACHN cells were transfected with this construct (miR-21 Sponge). DNA synthesis was measured as ^3^H-thymidine incorporation. Expression of miR-21 Sponge significantly inhibited DNA synthesis in ACHN cells (Fig. 2A and Fig. S4A). To confirm this observation, we performed proliferation assay by counting the number of cells after transfecting miR-21 Sponge. Expression of miR-21 Sponge suppressed cell growth (Fig. 2B and Fig. S4B).

Renal cell carcinoma is often highly metastatic. We tested whether miR-21 controls ACHN cell migration using trans-well chamber assay. Cells cultured in a serum-free medium on the top of the membrane migrated to the bottom of the membrane (Fig. 2C, panel a). Expression of miR-21 Sponge prevented the migration of ACHN cells (Fig. 2C and Fig. S4C). Quantification of these results shows significant inhibition of migration of renal cancer cells in response to miR-21 Sponge (Fig. 2D).

The initial step in metastasis consists of local invasion by cancer cells (intravasation). To examine the metastatic potential of ACHN renal cancer cells, we employed an invasion assay using collagen-embedded membranes in trans-wells. Vector-transfected ACHN cells showed marked invasion (Fig. 2E, panel a). Expression of miR-21 Sponge blocked the invasion of tumor cells (Fig. 2E and Fig. S4D). Quantification showed significant inhibition of invasion of ACHN cells by miR-21 Sponge (Fig. 2F).

### miR-21 Targets PTEN in Renal Cancer Cells

**Figure 3:**
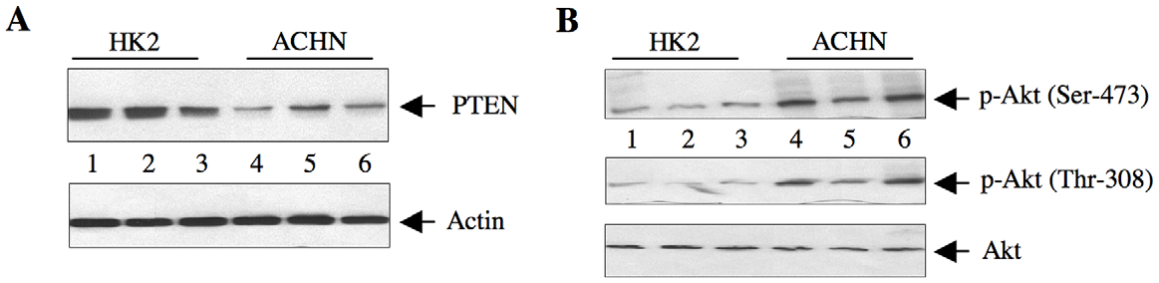
Expression of PTEN in HK2 and ACHN cells. (A) The lysates of cells from three independent wells of HK2 and ACHN cells were immunoblotted with PTEN and actin antibodies. (B) The same cell lysates were immunoblotted with phospho-Akt (Thr-308), phospho-Akt (Ser-473) and Akt antibodies.

Mutation in PTEN tumor suppressor gene or its reduced expression contribute to tumorigenesis and metastasis of many cancers [27], [28]. However, PTEN mutation is not frequently found in renal cell carcinoma [29]. PTEN has been validated to be a target of miR-21 [30]. We investigated the levels of PTEN in ACHN renal cancer cells. As shown in Fig. 3A, the abundance of PTEN in ACHN was significantly reduced as compared to that in HK2 cells. Identical results were obtained in Caki-2 renal carcinoma cell line (Fig. S5A). PTEN reduces the levels of PIP_3_, which controls the phosphorylation/activation of Akt [27], [31], [32]. Reduced PTEN levels in ACHN and Caki-2 cells were associated with increased phosphorylation of Akt at Thr-308 and Ser-473 (Fig. 3B and Fig. S5B), indicating its activation [32].

**Figure 4:**
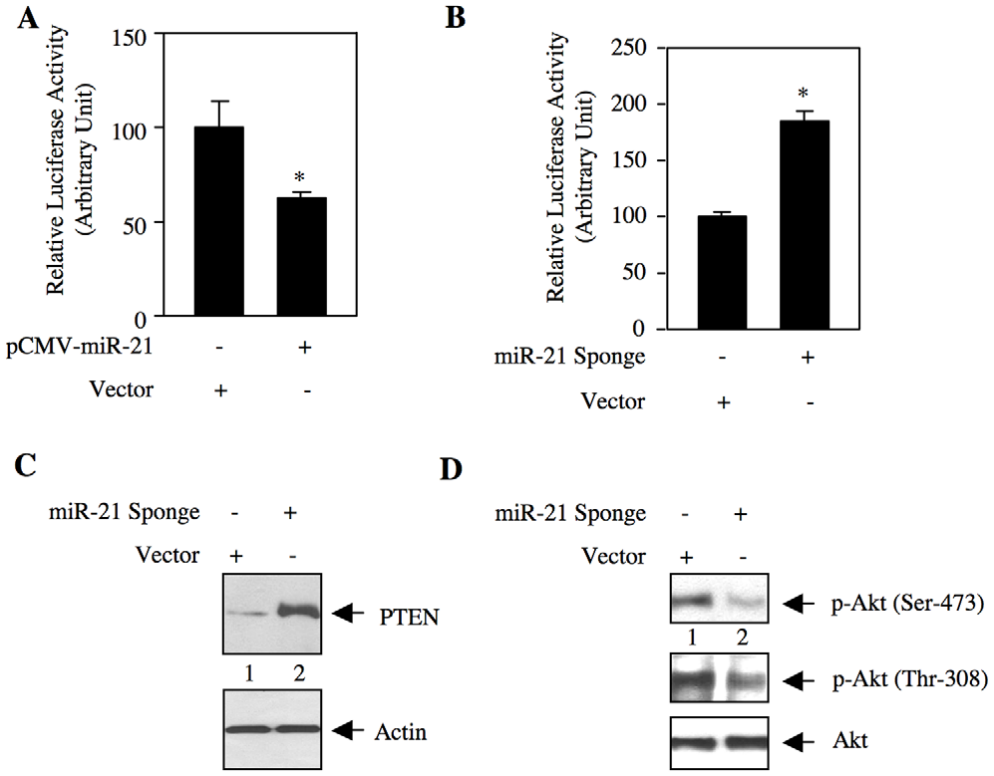
miR-21 regulates expression of PTEN in renal cancer cells. (A and B) ACHN cells were transfected with pCMV-miR-21 expression plasmid (A) or miR-21 Sponge (B) and vector along with PTEN 3′UTR-Luc reporter construct. The cell lysates were used to determine luciferase activity as described in the Materials and Methods. Mean ± SE of 6 measurements is shown. In panel A, *p  = 0.02 vs vector alone. In panel B, *p  = 0.002 vs vector alone. (C and D) Lysates of miR-21 Sponge-transfected ACHN cells were immunoblotted with PTEN and actin antibodies (panel C), and phospho-Akt (Ser-473), phospho-Akt (Thr-308) and Akt antibodies (panel D).

Next, we examined whether miR-21 targets PTEN. We used a reporter construct in which the 3′UTR of PTEN mRNA is cloned downstream of firefly luciferase gene (PTEN 3′UTR-Luc) [25]. ACHN cells were transfected with this reporter and vector or miR-21 expression plasmid. Expression of miR-21 significantly inhibited the reporter activity (Fig. 4A and Fig. S6A). To confirm this observation, we used miR-21 Sponge. Expression of miR-21 Sponge markedly increased the luciferase activity of PTEN 3′UTR-Luc (Fig. 4B and Fig. S6B). These results suggest that in renal cancer cells, PTEN may be a downstream target of miR-21. To test this, we examined PTEN protein level in miR-21 Sponge-transfected ACHN cells. miR-21 Sponge increased the abundance of PTEN (Fig. 4C and Fig. S6C), concomitant with decreased phosphorylation of Akt at Thr-308 and Ser-473 (Fig. 4D).

### miR-21 Regulates Renal Cancer Cell Proliferation and Migration via PTEN/Akt Axis

**Figure 5:**
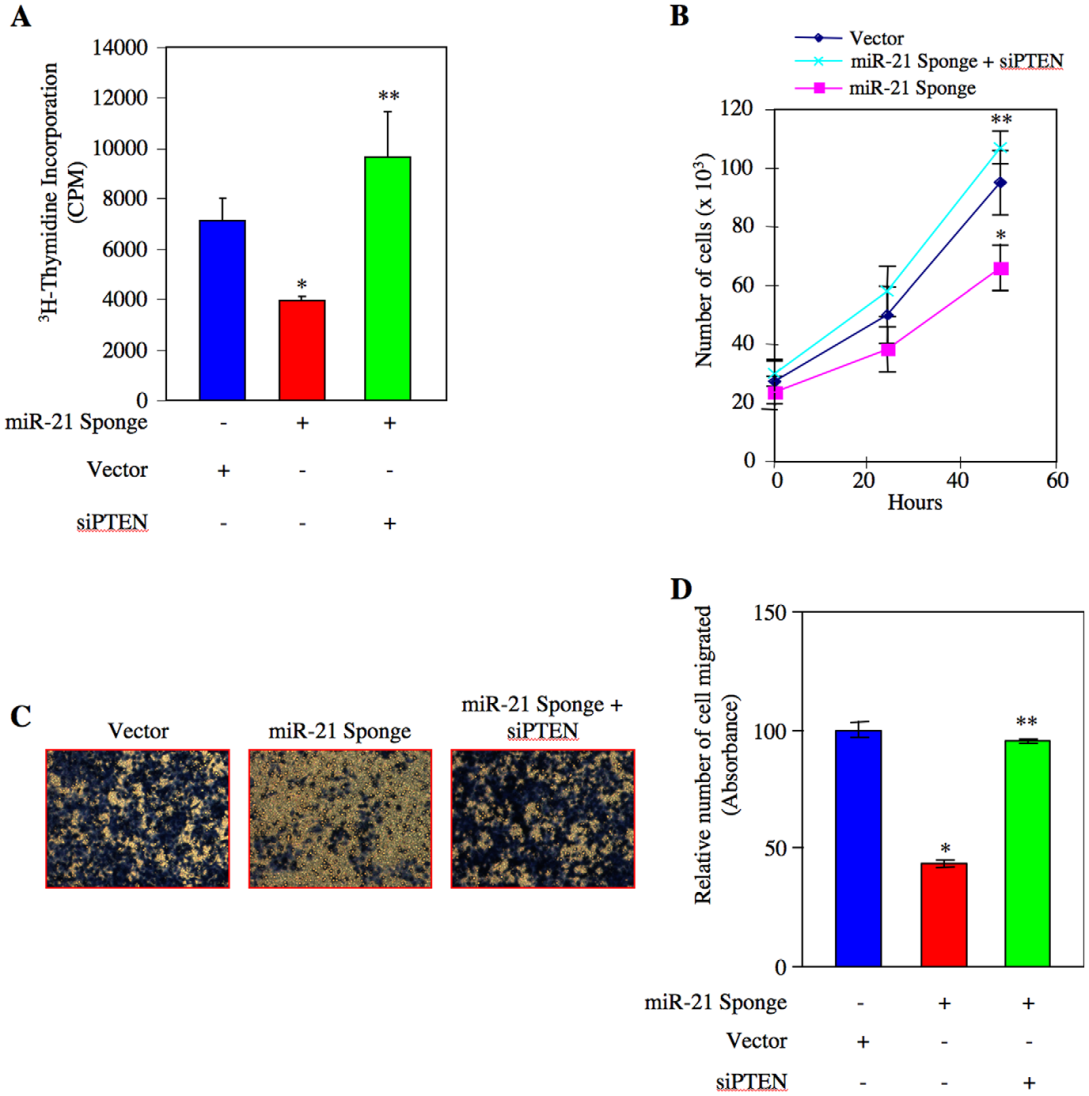
miR-21 targets PTEN to induce proliferation and migration of renal cancer cells. ACHN cells were transfected with miR-21 Sponge along with siRNA pools targeting PTEN mRNA as indicated. (A) ^3^H-thymidine incorporation was determined as described in the Materials and Methods [115]. Mean ± SE of 6 measurements is shown. *p<0.05 vs vector; **p<0.05 vs miR-21 Sponge-transfected cells. (B) Transfected cells were counted at indicated time periods. The symbols diamond, square and cross represent vector, miR-21 Sponge and miR-21 Sponge plus siRNA pool against PTEN, respectively. Mean ± SE of triplicate measurements is shown. *p<0.05 vs vector alone; **p<0.01 vs miR-21 Sponge alone. (C) Transfected cells were seeded onto membrane in trans-well chambers and the migrated cells were stained as described in the Materials and Methods [111]. (D) Stains from the membranes in panel C were eluted and absorbance at 590 nm was measured. Mean ± SE of 3 independent chambers is shown. *p<0.001 vs vector alone; **p<0.001 vs miR-21 Sponge.

Our results above suggest that miR-21 promotes Akt activation by decreasing PTEN levels in renal cancer cells (Fig. 4). In order to directly investigate the role of this signaling pathway in renal cancer cell proliferation, we transfected ACHN and Caki-2 cells with miR-21 Sponge and siRNAs targeting PTEN (siPTEN). As expected, miR-21 Sponge inhibited DNA synthesis (Fig. 5A and Fig. S7A). In contrast, transfection of siPTEN significantly prevented miR-21 Sponge-induced inhibition of DNA synthesis (Fig. 5A and Figs. S7A, S7B and S7C). Similarly, siPTEN reversed the decrease in proliferation of ACHN cells induced by miR-21 Sponge (Fig. 5B and Fig. S8). Next, we determined the effect of siPTEN on ACHN and Caki-2 cell migration. Expression of miR-21 Sponge decreased the migration of both these renal cancer cell lines. siPTEN significantly suppressed the inhibitory effect of miR-21 Sponge on cell migration (Figs. 5C, 5D and Figs. S9 and S10).

**Figure 6:**
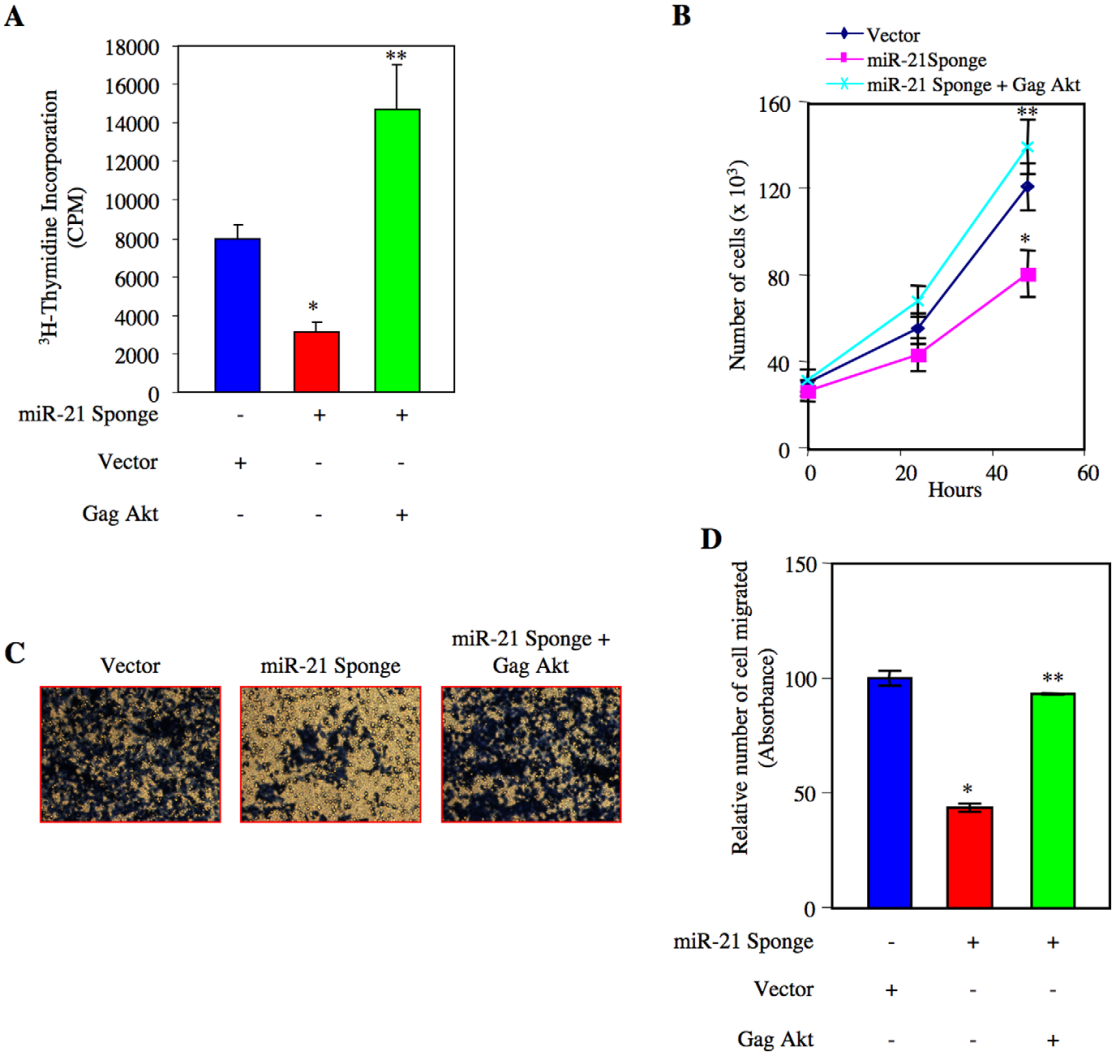
miR-21 stimulates Akt kinase to induce proliferation and migration of renal cancer cells. ACHN cells were transfected with miR-21 Sponge along with constitutively active Gag Akt plasmids as indicated. (A) ^3^H-thymidine incorporation was determined as described in the Materials and Methods [115]. Mean ± SE of 6 measurements is shown. *p<0.01 vs vector; **p<0.001 vs miR-21 Sponge alone. (B) Transfected cells were counted at indicated time periods. The symbols diamond, square and cross represent vector, miR-21 Sponge and miR-21 Sponge plus Gag Akt expression plasmids, respectively. *p<0.01 vs vector alone; **p<0.001 vs miR-21 Sponge alone. (C) Transfected cells were seeded onto membrane in trans-well chambers and the migrated cells were stained as described in the Materials and Methods [111]. (D) Stains from the membranes in panel C were eluted and absorbance was measured. Mean ± SE of 3 independent chambers is shown. *p<0.001 vs vector alone; **p<0.001 vs miR-21 Sponge.

The lipid phosphatase activity of PTEN is known to regulate the Akt phosphorylation [27]. We have shown above that reduced PTEN level in renal cancer cells is associated with increased phosphorylation of Akt (Fig. 3 and Fig. S5). Since miR-21 regulates PTEN protein level, which in turn activates Akt, we tested the role of this kinase in renal cancer cell proliferation in relation to miR-21. We transfected ACHN and Caki-2 cells with constitutively active Gag-Akt along with miR-21 Sponge [33]. Expression of Gag Akt significantly reversed the inhibition of DNA synthesis induced by miR-21 Sponge (Fig. 6A, Fig. S11A and Fig. S12). Expression of Gag Akt also reversed the cell proliferation by miR-21 Sponge (6B and Figs. S13A). Similarly, constitutively active Akt significantly prevented the inhibition of migration of ACHN cells in response to miR-21 Sponge (Figs. 6C, 6D and Fig. S13B). Identical results were obtained with Caki-2 renal cancer cells (Figs. S14A, S14B and S14C). These results indicate that miR-21 targets PTEN mRNA to suppress its protein expression, which leads to Akt-dependent proliferation and migration of renal cancer cells.

### miR-21 Modulates TORC1 Activity to Induce Renal Cancer Cell Proliferation and Migration

**Figure 7:**
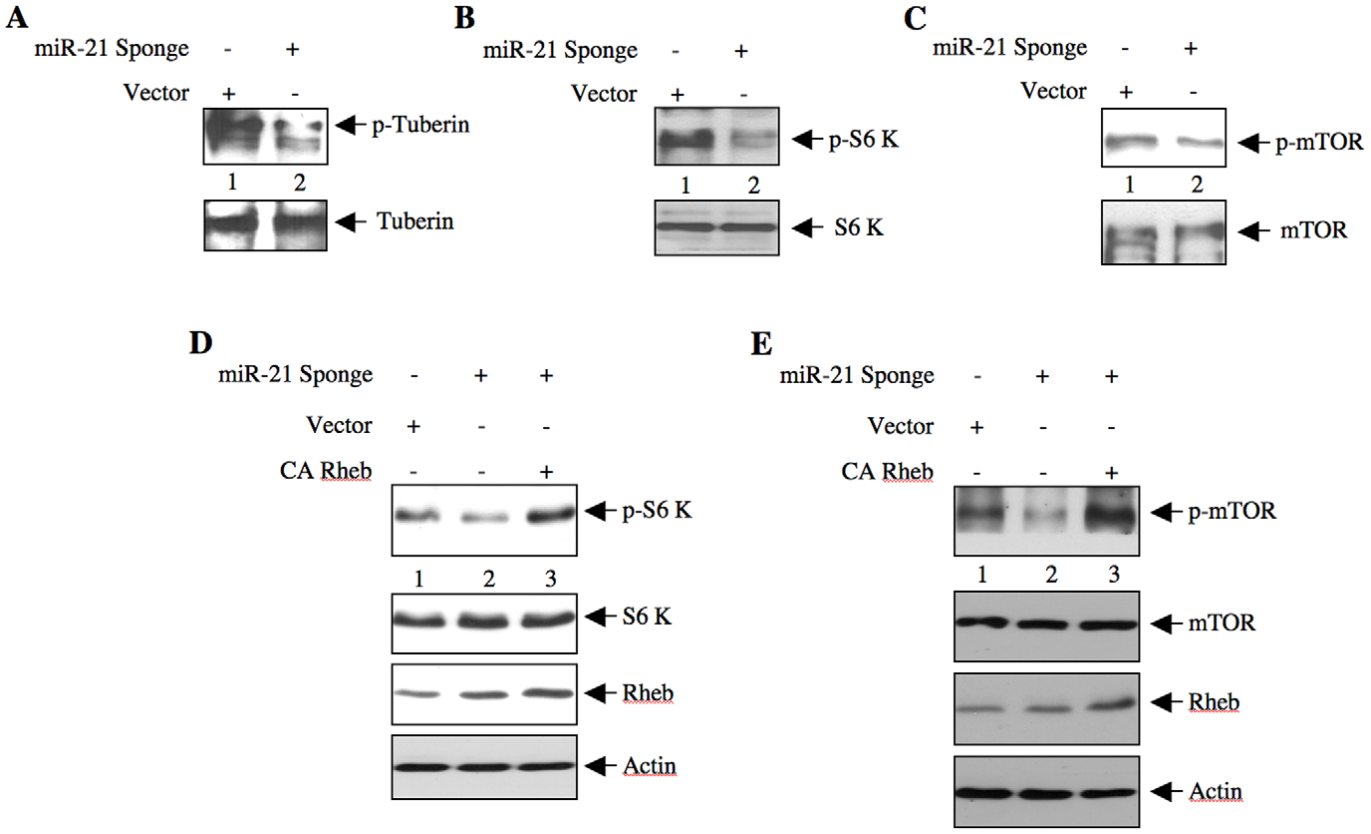
miR-21 regulates phosphorylation of tuberin and activation of mTOR in renal cancer cells. ACHN cells were transfected with miR-21 Sponge or vector. The cell lysates were immunoblotted with phospho-tuberin (Thr-1462), tuberin (panel A), phospho-S6 kinase (Thr-389), S6 kinase (panel B), phospho-mTOR (Ser-2448) and mTOR (panel C). miR-21 uses Rheb activation for TORC1 activity. ACHN cells were cotransfected with miR-21 Sponge and CA Rheb plasmid. The cell lysates were immunoblotted with phospho-S6 kinase (Thr-389), S6 kinase (panel D), phospho-mTOR (Ser-2448) and mTOR (panel E).

Activated Akt phosphorylates the tumor suppressor protein tuberin at Thr-1462 leading to its inactivation, and Rheb-mediated activation of TORC1 [34], [35], [36]. We have established that downregulation of PTEN due to increased miR-21 expression in renal cancer cells activates Akt (Fig. 3), which contributes to proliferation and migration of these cells (Figs. 5 and 6). We tested the role of miR-21 in tuberin phosphorylation. ACHN cells were transfected with miR-21 Sponge. Due to increased Akt activation, ACHN cells display enhanced phosphorylation of tuberin (Fig. 7A, lane 1). Expression of miR-21 Sponge inhibited the phosphorylation of tuberin (Fig. 7A and Fig. S15A). Similar to increased tuberin phosphorylation, ACHN cells showed augmented phosphorylation of S6 kinase at Thr-389 (Fig. 7B, lane 1), measured as a surrogate for TORC1 activity. miR-21 Sponge blocked S6 kinase phosphorylation (Fig. 7B). This reduction in S6 kinase phosphorylation by miR-21 Sponge was associated with inhibition of mTOR phosphorylation at Ser-2448 (Fig. 7C). These results suggest that miR-21 regulates TORC1 activity in the renal cancer cells. To examine whether miR-21 Sponge-dependent mTOR inhibition is due to inactivation of Rheb, we cotransfected ACHN cells with miR-21 Sponge and a constitutively active Rheb expression plasmid and the results were compared with that in miR-21 Sponge-transfected cells. mTOR activation was examined in the cell lysates. Expression of constitutively active Rheb reversed the inhibitory effect of miR-21 Sponge on S6 kinase phosphorylation (Fig. 7D and Fig. S15B). Similarly, the reduced phosphorylation of mTOR by miR-21 Sponge was also prevented by constitutively active Rheb (Fig. 7E and Fig. S15C). These results conclusively demonstrate that miR-21-mediated phosphorylation/inactivation of tuberin activates Rheb to increase mTOR activity.

**Figure 8:**
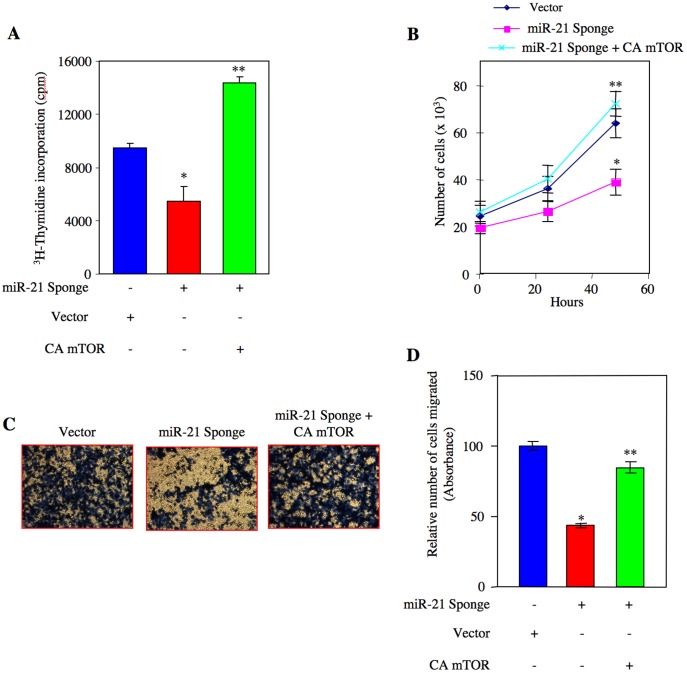
miR-21 regulates proliferation and migration of renal cancer cells through activation of TORC1. ACHN cells were transfected with miR-21 Sponge along with constitutively active mTOR plasmids as indicated. (A) ^3^H-thymidine incorporation was determined as described in the Materials and Methods [115]. Mean ± SE of 6 measurements is shown. *p<0.001 vs vector; **p<0.001 vs miR-21 Sponge alone. (B) Transfected cells were counted at indicated time periods. The symbols diamond, square and cross represent vector, miR-21 Sponge and miR-21 Sponge plus constitutively active (CA) mTOR expression plasmids, respectively. *p<0.01 vs vector alone; **p<0.001 vs miR-21 Sponge alone. (C) Transfected cells were seeded onto membrane in trans-well chambers and the migrated cells were stained as described in the Materials and Methods [111]. (D) Stains from the membranes in panel C were eluted and absorbance at 590 nm was measured. Mean ± SE of 3 independent chambers is shown. *p<0.001 vs vector alone; **p<0.001 vs miR-21 Sponge.

To elucidate whether miR-21-activated TORC1 contributes to proliferation of renal cancer cells, we cotransfected ACHN and Caki-2 cells with miR-21 Sponge and a constitutively active mTOR expression vector. ^3^H-thymidine incorporation was determined. Expression of constitutively active mTOR significantly prevented the inhibition of DNA synthesis by miR-21 Sponge (Fig. 8A, Fig. S16 and Fig. S17). Similarly, constitutively active mTOR inhibited the miR-21 Sponge-mediated decrease in ACHN cell proliferation (Fig. 8B and Fig. S18A). Next we examined the effect of constitutively active mTOR on renal cancer cell migration. miR-21 Sponge abolished migration of ACHN and Caki-2 cells (Fig. 8C, Fig. S18B and Fig. S19). However, coexpression of constitutively active mTOR with miR-21 Sponge significantly reversed the inhibition in migration of both renal cancer cell lines in response to miR-21 Sponge (Figs. 8C, 8D and Fig. S19). Taken together these results demonstrate that miR-21 contributes to renal cancer cell proliferation and migration via TORC1.

## Discussion

**Figure 9:**
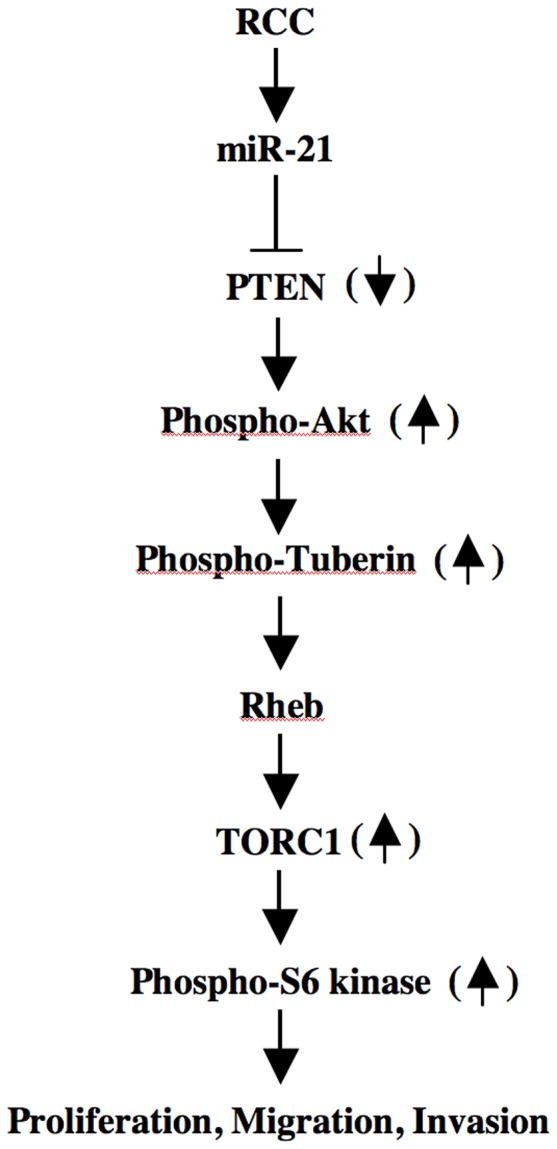
Schematic demonstrating our results. Enhanced miR-21 attenuates PTEN protein levels, resulting in activation of Akt, which inactivates tuberin to increase TORC1 activity leading to proliferation, migration and invasion of renal cancer cells.

In renal cancer cells, we provide evidence for the expression of pri-miR-21 to produce pre- and mature miR-21, which contributes to proliferation and migration/invasion. We show an inverse correlation between miR-21 levels and PTEN abundance. We demonstrate that miR-21-sensitive PTEN regulates proliferation and migration of renal cancer cells via activation of Akt. Finally, we establish a role for miR-21-stimulated TORC1 in renal cancer cell proliferation and migration (Fig. 9).

Altered gene expression controls signaling networks to regulate the biological outputs, which contribute to cell transformation and metastasis. Although transcriptional regulation of genes to produce mRNAs mediates a substantial contribution to oncogenesis, recent discovery of miRNAs playing a role similar to DNA binding transcription factors is established in regulating expression of target genes by post-transcriptional mechanism. Many miRNAs have been identified and characterized as tumor promoters as well as tumor suppressors. For example, the ubiquitously expressed miRNA, miR-21, is frequently upregulated in many cancers including breast, lung, neuroblastoma, glioblastoma, leukemia, gastric cancer, cholangiocarcinoma, colon cancer and hepatocellular cancer [37], [38], [39], [40], [41], [42]. However, information about the functional role of miR-21 is available only from *in vitro* studies. Only recently the *in vivo* role of miR-21 in development of cancer has been demonstrated in pre-B-cell lymphoma and non-small cell lung carcinoma. In both these cancers, expression of miR-21 is significantly increased [43], [44], [45]. Using mouse models of overexpression as well as deletion of miR-21, Medina et al and Hatley et al recently showed a highly specific role of this miRNA in the development of pre-B-cell lymphoma and non-small cell lung carcinoma, respectively [46], [47].

miR-21 regulates proliferation and mitochondrial apoptotic pathways controlled by tumor suppressor proteins, cell cycle regulators and growth factors [48], [49]. miR-21 is expressed abundantly in kidney proximal tubules [50]. In animal models, increased expression of miR-21 is associated with fibrotic disorders of kidney and renal ischemic injury [51], [52], [53], [54]. Recently, we have reported increased expression of miR-21 in a model of type 1 diabetic nephropathy [25]. We showed that miR-21 regulates hypertrophy of proximal tubular epithelial cells, indicating the pathological role of this miRNA in renal diseases [25]. Similarly, miRNA profiling studies identified increased expression of miR-21 in both clear cell and papillary renal cell carcinomas [14], [19], [55]. In contrast, in a recent microarray analysis of 30 grades 1 and 2 clear cell renal carcinoma tissue, miR-21 was reported to be downregulated by more than 2-fold [56]. Interestingly, a weak reciprocal correlation was detected between survival of patients with clear cell renal carcinoma and miR-21 expression [55]. In this report downregulation of miR-21 was found in VHL deficient RCC4 renal cancer cell line [55]. Reconstitution of VHL resulted in increased expression of miR-21 in a Hifα-independent manner. It should be noted that 42% of the clear cell tumors show VHL mutation and 10% of the tumors carry methylation in the VHL promoter [57], [58], [59]. In the present study, we show a significant increase in miR-21 expression in the VHL positive ACHN and Caki-2 renal cancer cells compared to normal proximal tubular epithelial cells (Fig. 1 and Fig. S2) [60]. Furthermore, our results demonstrate significantly enhanced miR-21 expression in 100% of grade 2 and grade 3 renal tumor tissues compared to matched control tissue (Fig. S1). Additionally, using a VHL negative renal cancer cell line, 786-O, we found significantly increased expression of miR-21 as compared to HK2 proximal tubular epithelial cells (Fig. S20). These results demonstrate that irrespective of VHL status, renal cancer cells express high levels of miR-21.

Metastatic renal carcinoma cells display increased TGFβ-dependent Smad 2/3 signaling [61]. Recently a role of TGFβ in increasing the levels of mature miR-21 has been reported [62]. These authors described a transcription-independent function of TGFβ-specific Smad proteins, which are recruited into the RNase III Drosha through interaction with RNA helicase p68. This microprocessor complex recruits pri-miR-21 to facilitate production of pre-miR-21, thus increasing the levels of mature miR-21 [62]. This study did not find any increase in pri-miR-21 in response to TGFβ. More recently, the same group reported the presence of TGFβ-specific Smad binding element in the stem region of pri-miR-21, which recruits Smad directly to the Drosha microprocessor/pri-miR-21 complex for increased processing to produce pre-miR-21, providing another mechanism for more miR-21 production in response to TGFβ [20]. In contrast to these observations, we found increased expression of pri-miR-21 in the metastatic ACHN renal cancer cells (Fig. 1). In addition, we identified increased transcription of miR-21 gene by constitutively active NFκB present in these cells (Fig. 1). This observation provides a mechanism for enhanced levels of miR-21 detected in renal carcinoma cells. Furthermore, in all of grade 2 and grade 3 renal tumor samples examined, we detected increased levels of pri-miR-21 (data not shown). Thus in renal cell carcinomas, our results demonstrate the existence of a regulatory mechanism for increased mature miR-21 expression through increased transcriptional activation of pri-miR-21 locus.

Since miRNAs essentially downregulate the expression of target proteins, their increased expression in relation to tumorigenesis and metastasis may possibly counteract the negative regulatory mechanisms that operate in the proliferation/invasion signaling pathways. We found enhanced levels of miR-21 in the renal cancer cells. Inhibition of miR-21 significantly abrogated DNA synthesis, proliferation, migration and invasion of these cells. These results are in line with the recent demonstration of a positive correlation between miR-21 levels and tumor size in patients with renal carcinoma [55].

PI 3 kinase/Akt signaling pathway contributes to carcinogenesis and metastasis of various organs including kidney. Activation of these enzymes occurs due to increased growth factor receptor activation as well as gain of function mutations in these two enzymes. In fact 3000 different somatic mutations have been identified in the p110α catalytic subunit of PI 3 kinase in various cancers [63]. However, its mutation in the renal cancer is extremely rare. Similarly, although activating mutation in Akt occurs in various cancers, it has not been reported in renal carcinoma [64]. Mutation in the PTEN gene, which codes for a protein phosphatase as well as a lipid phosphatase and regulates Akt activity, has been identified in many cancers [65]. Similar to PI 3 kinase and Akt, PTEN mutation is rare in renal cell carcinoma. However, 25% of clear cell renal carcinomas display about 25% reduction in PTEN protein levels [66], [67]. PTEN is known to be transcriptionally downregulated in many cancer cells, e.g., by NFκB-mediated transcriptional repression [68]. PTEN is also regulated at post-translational level by phosphorylation, acetylation, oxidation, ubiquitinylation and proteasomal degradation [69]. Furthermore, PTEN expression is lost in many human tumors due to its promoter methylation [70]. Although PTEN promoter methylation was detected in the renal cancer cells, it did not affect PTEN transcription [71]. An additional mode of post-transcriptional regulation of PTEN protein expression includes a host of miRNAs, including miR-21 [72], [73]. The half lives of mature miRNAs are controlled by extracellular signals and cell cycle status [74]. In renal tumor tissue and in metastatic ACHN and Caki-2 renal cancer cells, we demonstrate constitutive increase in the levels of mature miR-21 (Fig.1 and Fig. S2). Furthermore, in renal cancer cells, a reciprocal relationship exists between miR-21 and PTEN protein expression (Fig. 3 and Fig. S5). Also, we validated functional activity of miR-21 in ACHN cells in negatively maintaining the PTEN protein abundance (Fig. 4). PTEN prevents cell cycle arrest and apoptosis in a cell-specific manner and also suppresses cell invasion [65], [75]. Our results demonstrate that inhibition of PTEN reversed miR-21 Sponge-mediated decrease in DNA synthesis and proliferation (Figs. 5A, 5B and Fig. S7A). These data indicate that miR-21 regulates cell cycle progression of renal cancer cells via PTEN. Importantly, miR-21-targeted PTEN contributes to the migration of renal cancer cells (Figs. 5C, 5D and Fig. S10).

Along with its lipid phosphatase activity, PTEN also possesses phosphatase activity towards specific tyrosine phosphorylated proteins including focal adhesion kinase, c-Src and platelet-derived growth factor receptor [76], [77], [78]. The protein phosphatase activity of PTEN is required for cell proliferation, migration and invasion [78], [79], [80]. Our results showing a role for miR-21-targeted PTEN in renal cancer cell migration do not discriminate between these two enzymatic activities of PTEN (Fig. 5 and Fig. S10). Furthermore, a study in *Drosophila* showed that a PH-domain mutant of Akt, which does not bind PIP_3_ but retains 80% of wild type kinase activity, reverses the lethality caused by absence of PTEN [81]. These results conclusively demonstrate that the only function of PTEN loss is to translocate Akt to the membrane to enhance its kinase activity. In our study, we used constitutively active Gag Akt, which localizes to the plasma membrane and bypasses the PTEN-mediated regulatory loop [33]. Our results using constitutively active Gag Akt to prevent the inhibitory action of miR-21 Sponge on DNA synthesis, proliferation and migration of ACHN and Caki-2 cells demonstrate directly the requirement of PIP_3_ phosphatase activity of PTEN for these biological functions in renal cancer cells (Fig. 6 and Figs. S12, S14). Thus, our results demonstrate that miR-21 predominantly targets the lipid phosphatase activity of PTEN to regulate Akt kinase in favor of renal cancer cell proliferation and migration.

Reduced expression of PTEN resulting in Akt activation is associated with cancer progression in many organs including breast tumor [82], [83]. In addition, miR-21 is upregulated in many solid tumors [18]. To test the generality of our observation, we examined the involvement of miR-21 in regulation of PTEN-mediated proliferation of BT-20 human breast cancer cells. Expression of miR-21 in these cells was significantly increased as compared to the MCF10A normal breast epithelial cells (Fig. S21). Expression of miR-21 Sponge markedly inhibited the proliferation of BT-20 cells (Fig. S22A). Cotransfection of siPTEN reversed the inhibition of proliferation induced by miR-21 Sponge (Fig. S22A, S22B). Similarly, siPTEN prevented the reduction of BT-20 breast cancer cell migration induced by miR-21 Sponge (Figs. S23A, S23B). Next, we determined the involvement of Akt in the effect of miR-21 Sponge in these breast cancer cells. Similar to the results observed in renal cancer cells, expression of constitutively active Gag-Akt reversed the inhibitory effect of miR-21 Sponge on BT-20 cell proliferation and migration (Figs. S24 and S25). These results indicate that miR-21 targeting of PTEN to activate Akt contributes to proliferation and migration of other cancer cells along with the renal cancer cells.

PTEN heterozygous mice produce noninvasive prostate cancer with 50% penetrence, while mice with p27 homozygous deletion do not develop prostate cancer [84], [85]. Interestingly, all PTEN^+/−^p27^−/−^ mice develop prostate cancer [85]. Similarly, in a recent study absence of both PTEN and the cell cycle inhibitor p27 was shown to be associated with renal cancer progression [86]. Furthermore, in these patients phosphorylation of ribosomal protein s6 was significantly correlated with tumor stage and grade [86]. Since s6 is a substrate of TORC1, these results predict high activity of this kinase complex in renal cancer cells.

mTOR exists in two independent complexes each containing shared and unshared protein subunits with non-overlapping substrate specificity [87], [88], [89], [90]. Both TORC1 and TORC2 regulate cell proliferation and apoptosis of tumor cells by regulating distinct kinases [88], [91], [92]. For example, TORC1 augments inactivating phosphorylation of the translational repressors 4EBPs and activating phosphorylation of translational activator S6 kinase [91]. Increased mRNA translation is a prerequisite for cell proliferation [93]. On the other hand, TORC2 phosphorylates Akt at the hydrophobic motif site Ser-473 [92]. Akt plays a pivotal role in mTOR-mediated signaling as it acts both upstream of TORC1 and downstream of TORC2 [91]. Thus Akt phosphorylates tuberin at Thr-1462 to inactivate its suppressive action on Rheb, resulting in TORC1 activation [35], [94]. Inactivation of endogenous miR-21 in renal cancer cells blocked phosphorylation of tuberin (Fig. 7A). This inhibition of tuberin phosphorylation was associated with decreased phosphorylations of S6 kinase and mTOR (Fig. 7B and 7C) [90], [91]. Furthermore, our results demonstrate that miR-21 utilizes Rheb to increase TORC1 activity (Figs. 7D and 7E). Expression of constitutively active mTOR, which increases only TORC1 activity without affecting TORC2, reversed the inhibitory effect of miR-21 Sponge on DNA synthesis and proliferation (Figs. 8A, 8B and Fig. S17) [95]. These results thus indicate that in renal cancer cells, miR-21 regulates the TORC1 activity by acting upstream of Akt, presumably through targeting PTEN. PTEN as a PIP_3_ phosphatase maintains the level of this lipid in cells and regulates TORC2 activity [96], [97]. In fact we demonstrate inhibition of phosphorylation of Akt at the TORC2 site Ser-473 by miR-21 Sponge (Fig. 4D). Thus, our results show that miR-21 acts through both TORC1 and TORC2.

TORC2 regulation of Rho GTPase, paxillin phosphorylation, cytoskeletal organization and cell migration was initially reported [89], [98]. More recently TORC1-mediated S6 kinase pathway has been shown to regulate phosphorylation of paxillin, p130^CAS^ and focal adhesion kinase, which contribute to lamillapodia formation and cell migration [99], [100]. Rapamycin, which predominantly inhibits TORC1 activity, abrogates invasion of normal as well as many cancer cells *in vitro* and metastasis of implanted tumor cells in mouse models, including pulmonary metastasis of human renal cancer cells [101], [102]. In the present study, we show that expression of constitutively active TORC1 reversed the inhibition of renal cancer cell migration in response to miR-21 Sponge (Figs. 8C and 8D). Thus our results demonstrate a role of miR-21-induced TORC1 activity in renal cancer cell invasion.

Although many rapalogs including temsirolimus and everolimus with increased solubility and bioavailability are approved for treatment of metastatic renal cancer, clinical trials showed limited efficacy [103], [104], [105], [106]. This is thought to be due to the release of the negative feedback loop involving IGF-1 receptor signaling on phosphorylation of Akt at Ser-473 by TORC2 [107], [108]. In fact use of rapamycin in a small clinical trial with 15 recurrent glioblastoma patients lacking PTEN expression showed increased phosphorylation of Akt at Ser-473 in 50% of the patients, indicating the importance of relief of the negative feed back loop *in vivo* [109]. In our study here, we demonstrate reduced PTEN levels in renal cancer cell due to increased miR-21 expression, which causes TORC1-mediated proliferation and invasion. Quenching of miR-21 blocked proliferation and migration, which result from attenuation of Akt phosphorylation at Thr-308 and Ser-473. Thus, this strategy of miR-21 inhibition does not evoke the release of negative feedback loop in these cells. Therefore, use of anti-miR-21 in preclinical model of renal cell carcinoma holds promise to provide a therapeutic option for this devastating cancer.

## Materials and Methods

### Materials

β-actin antibody, phenylmethylsulfonylfluoride, Na_3_VO_4_, NP-40 and protease inhibitor cocktail were obtained from Sigma, St. Louis, MO. Antibodies against phospho-Akt (Ser-473 and Thr-308), phospho-S6 kinase (Thr-389), phospho-tuberin (Thr-1462), phospho-mTOR (Ser-2448), phospho-p65 (Ser-536), Akt, S6 kinase, mTOR and tuberin were purchased from Cell Signaling, Boston, MA. p65 and PTEN antibodies and siRNA pool of three oligonucleotides against human PTEN were obtained from Santa Cruz, Delaware, CA. Trans-well migration chambers were purchased from Greiner Bioone, Monroe, NC. Invasion chambers were obtained from Millipore, MA. Fugene-HD transfection reagent was purchased from Roche Molecular Biology, Indianapolis, IN. TRIZol reagent for RNA isolation was purchased from Invitrogen, Carlsbad, CA. RT^2^ real-time SYBR green/ROX PCR master mix, RT^2^ miRNA first strand kit, GAPDH RT-PCR primers for human and primers for detection of mature miR-21 were obtained from SuperArray Biosciences, Frederick, MD. U6 primers (for normalization) were obtained from Ambion, Austin, TX. Luciferase Reporter Assay System kit was purchased from Promega, Madison, WI. ^3^H-thymidine was purchased from PerkinElmer, Boston, MA. pCMV-miR-21 expression plasmid, PTEN 3′ UTR-Luc reporter plasmid and miR-21 Sponge vector have been described previously [25]. Constitutively active (CA) Rheb (phage-CMV-Rheb (S16H) was obtained from Addgene. Constitutively active mTOR expression vector was provided by Dr. Tatsuya Maeda, The University of Tokyo, Japan [95]. miR-21 promoter driven luciferase reporter (-332 to +1957 bp) plasmid (miR-21-Luc) was a kind gift from Dr. X-M Chen, Creighton University Medical Center, Nebraska [21]. The constitutively active Akt (Gag Akt) was a kind gift from Dr. J. Downward, Signal Transduction Laboratory, London, UK [33].

### Human Tumor Specimens/Ethics Statement

Tumor samples and normal corresponding tissue from patients with renal cancer were obtained from the Department of Urology at the University of Texas Health Science Center at San Antonio. The collection and handling of human samples was carried out according to a protocol approved by the University of Texas Health Science Center at San Antonio, Institutional Review Board (IRB protocol #HSC 20070777N). This is a “Non Human/NonResearch” protocol. Tissue is collected under non-identifiers from Pathology using "unwanted tissue". Therefore, patient consent is not necessary. The tumors for this study were histologically classified as clear cell renal carcinoma and staged according to the TNM classification.

### Cell Culture

The ACHN renal carcinoma cell line was purchased from American Type Culture Collection, Manassas, VA, and grown in RPMI 1640 medium containing 10% fetal bovine serum and penicillin/streptomycin. Caki-2 cells were kind gift of Dr. Sunil Sudarsan, Department of Urology, The University of Texas Health Science Center at San Antonio. These cells were grown in DMEM containing low glucose with 10% fetal bovine serum. HK2 normal human proximal tubular cells were grown in DMEM/F12 in the presence of 10% fetal bovine serum [110] BT-20 human breast cancer cells have been described previously [111].

### Real Time Quantitative RT-PCR (qRT-PCR)

Total RNA was prepared from HK2 and ACHN cells and from human tissue using TRIZol reagent as described previously [25], [68]. One µg of RNA was used to synthesize cDNA using RT^2^ miRNA first strand kit according to the manufacturer’s instructions. qRT-PCR was performed using a real-time PCR machine (7900HT, Applied Biosystems). Each sample was analyzed in duplicate. PCR conditions for pre-miR-21 were: 94°C for 10 minutes, followed by 40 cycles at 94°C for 30 seconds, 56°C for 30 seconds, 72°C for 40 seconds. The primers used for detection of pre-miR-21 are as follows: Forward primer: 5′-TGTCGGGTAGCTTATCAGAC-3′; Reverse primer: 5′-TTCAGACAGCCCATCGACTG-3′. Primers for pri-miR-21 are: Forward primer: 5′- ACAGGCCAGAAATGCCTGGG-3′; Reverse primer: 5′- GATGGTCAGATGAAAGATAC-3′. For mature miR-21, qRT-PCR primer sets for hsa-mir-21 (Superarray) were used according to the manufacturer’s protocol. The PCR conditions for amplifying pri-miR-21 were identical to those for pre-miR-21 except annealing was performed at 54°C. mirVana qRT-PCR primer sets for U6 (Ambion) were used for normalization. Data analyses were done by the comparative Ct method as described previously [25].

### Immunoblotting

Cells were lysed in RIPA buffer (20 mM Tris–HCl, pH 7.5, 150 mM NaCl, 5 mM EDTA, 1 mM Na_3_VO_4_, 1 mM PMSF, 0.1% protease inhibitor cocktail and 1% NP-40) at 4°C for half an hour as described previously [112], [113], [114]. The cell extracts were centrifuged at 10,000×g for 20 min at 4°C. Protein was estimated in the supernatant using BioRad reagent. Equal amounts of cell lysates were separated by SDS polyacrylamide gel electrophoresis and transferred to PVDF membrane. Proteins present in the membrane were immunoblotted with the indicated antibodies as described previously [25], [113], [114].

### DNA Synthesis and Proliferation Assay

Eighteen hours post-transfection, cells were serum deprived for 24 hours and incubated with ^3^H-thymidine for 18 hours. ^3^H-thymidine incorporation was determined as a measure of DNA synthesis as described previously [115]. For proliferation assay, eighteen hours post-transfection the cells were serum deprived for indicated time periods. The starting time of serum free medium was considered as time zero. The cells were trypsinized and counted in a hemocytometer as described [115].

### Transient Transfection

The cells were transfected with the indicated plasmids using Fugene HD as described previously [25], [111], [113], [114], [116].

### Migration and Invasion Assays

ACHN cells were transfected with vector or miR-21 Sponge plasmids. Both migration and invasion assays were performed essentially as described previously [111]. Briefly, 25×10^4^ ACHN cells were seeded in trans-well chambers containing a membrane with 8 µm pore size. The migration chambers placed in a 24-well plate were incubated for 14 hours at 37°C. The migrated cells were stained with the reagent using a kit and photographed followed by elution of the stains according to the vendor’s instruction. The absorbance of the eluted stain was measured at 590 nm and used arbitrarily as a measure of number of cells migrated. For the invasion assay, the cells were seeded in trans-well chambers with a membrane embedded with collagen and invasion measured as described above.

### Luciferase Assay

The cells were transfected with the miR-21-Luc reporter along with Renilla null reporter plasmid. Luciferase activities were determined in the cell lysate using a dual luciferase assay kit as described previously [68], [116]. Mean ± SE of triplicate measurements are presented as ratio of firefly and Renilla luciferase activity as described previously [68], [113], [114], [116], [117].

### Statistics

The significance of the data was analyzed by paired t-test. Where necessary ANOVA followed by Student–Newman–Keuls analysis was used as described previously [25], [113], [114], [118]. A p value less than 0.05 was considered as significant.

## Supporting Information

Figure S1 **Expression of mature miR-21 in Grade 2 and Grade 3 clear cell renal carcinomas.** Total RNAs from three renal tumor samples and from the normal portion of three kidneys were used for real time qRT-PCR to detect mature miR-21 as described in the text. The expression levels were normalized to U6. Each panel represents one subject. N, normal tissues; T, tumor tissues.
(TIF)

Figure S2 **Expression of miR-21 in Caki-2 renal cancer cells.** Total RNA from HK2 normal proximal tubular epithelial cells and Caki-2 cells was used for real time qRT-PCR to detect mature miR-21 as described in the text. The expression levels were normalized to U6. Mean ± SE of quadruplicate measurements is shown. *p  = 0.03 vs HK2.
(TIF)

Figure S3 **Structure of miR-21 Sponge expression of plasmid.** Consecutive 7 copies of anti-miR-21 sequence with a bulge in each were introduced downstream of green fluorescence protein (GFP) cDNA. Sequence of the anti-miR-21 bulge is shown at the bottom. The GFP sequence is driven by RNA Pol II from the human cytomegalovirus (CMV) early promoter.
(TIF)

Figure S4 Expression of miR-21 Sponge for the results described in Fig. 2A – 2F. ACHN cells were transfected with miR-21 Sponge or vector plasmids as indicated in the Figure 2. Total RNAs were used in RT-PCR for the detection of GFP mRNA, which serves as the surrogate for the expression of miR-21 neutralizing “Sponge” sequence. Detection of GAPDH mRNA was used as control. Panels A and B represent data for Fig. 2A and 2B respectively. Panels C represents data for Fig. 2C and 2D. Panel D shows data for Fig. 2E and 2F, respectively.
(TIF)

Figure S5 **Expression of PTEN and activation of Akt in Caki-2 renal cancer cells.** (A) Lysates of HK2 proximal tubular epithelial cells and Caki-2 cells were immunoblotted with PTEN and actin antibodies. (B) The same lyssates were immunoblotted with phospho-Akt (Ser-473 and Thr-308) and Akt antibodies as indicated.
(TIF)

Figure S6 (A) Expression of mature miR-21 for the results described in Fig. 4A in the text. ACHN cells were transfected with CMV-miR-21 or Vector. The total RNAs were used to detect mature miR-21. The level of miR-21 was corrected for U6 RNA expression. (B) Expression of miR-21 Sponge for the results presented in Fig. 4B. (C) Expression of miR-21 Sponge for the results presented in Figs. 4C and 4D. Total RNAs were used to detect GFP mRNA and GAPDH as described in the Fig. S4.
(TIF)

Figure S7 (A) Downregulation of PTEN reversed mir-21 Sponge-induced inhibition of DNA synthesis in Caki-2 renal cancer cells.^3^H-thymidine incorporation was used as a measure of DNA synthesis as described in the legend of Fig. 2A. Mean ± SE of 6 measurements is shown. *p<0.05 vs control; **p<0.01 vs miR-21 Sponge. (B) Expression of miR-21 Sponge and PTEN for the results described in panel A. Total RNAs and cell lysates were used from miR-21 Sponge and PTEN siRNA-transfected Caki-2 cells. GFP mRNA and GAPDH were detected as described in the Supplemental Fig. S4. Cell lysates were immunoblotted with PTEN and actin antibodies. (C) Expression of miR-21 Sponge and PTEN for the results described in Fig. 5A. Total RNAs and cell lysates were used from miR-21 Sponge and PTEN siRNA-transfected ACHN cells. GFP and GAPDH mRNAs were detected as described in the Supplemental Fig. S4. Cell lysates were immunoblotted with PTEN and actin antibodies.
(TIF)

Figure S8 **Expression of miR-21 Sponge and PTEN for the results described in** **Fig. 5B** **.** Expression of GFP and GAPDH mRNAs and PTEN and actin proteins were determined as described in the legend of Fig. S7C. Expression of miR-21 Sponge for the results described in Figs. 5C and 5D. Total RNAs were used to detect GFP and GAPDH and cell lysates were used for immunoblotting with PTEN and actin antibodies.
(TIF)

Figure S9 **Expression of miR-21 Sponge for the results described in** **Figs. 5C and 5D** **.** Total RNAs were used to detect GFP and GAPDH and cell lysates were used for immunoblotting with PTEN and actin antibodies.
(TIF)

Figure S10 (A) Downregulation of PTEN reversed mir-21 Sponge-induced inhibition of migration of Caki-2 renal cancer cells. Caki-2 cells were transfected either with miR-21 Sponge alone or along with siRNAs against PTEN. Migration of the transfected cells were measured as described in the legend of Fig. 2C. (B) The absorbance of the stain of the migrated cells in panel A was determined. Mean ± SE of 3 measurements is shown. *p<0.001 vs control; **p<0.001 vs miR-21 Sponge. (C) Expression of miR-21 Sponge and PTEN for the results described in panels A and B. Total RNAs and cell lysates were prepared from Caki-2 cells plated independently. GFP mRNA was detected as a surrogate for miR-21 Sponge expression. GAPDH was used as control. Cell lysates were immunoblotted with PTEN and actin antibodies.
(TIF)

Figure S11 **Expression of miR-21 Sponge and Akt for the results described in** **Fig. 6A** **.** Total RNAs and cell lysates were used from miR-21 Sponge and Gag Akt-transfected ACHN cells. GFP mRNA and GAPDH were detected.
(TIF)

Figure S12 (A) Expression of constitutively active Gag-Akt reversed mir-21 Sponge-induced inhibition of DNA synthesis in Caki-2 renal cancer cells.^3^H-thymidine incorporation was used as a measure of DNA synthesis as described in the legend of Fig. 2A. Mean ± SE of 6 measurements is shown. *p<0.01 vs control; **p<0.001 vs miR-21 Sponge. (B) Expression of miR-21 Sponge and Akt for the results described in panel A. Total RNAs and cell lysates were used from miR-21 Sponge and Gag-Akt-transfected Caki-2 cells. GFP mRNA was used as a surrogate for miR-21 epxression. Expression of GAPDH mRNA was used as control. Cell lysates were immunoblotted with Akt and actin antibodies.
(TIF)

Figure S13 (A) Expression of miR-21 Sponge and Gag Akt for the results described in Fig. 6B. Total RNAs and cell lysates were used from miR-21 Sponge and Gag-Akt-transfectedACHNcells. GFP mRNA was used as a surrogate for miR-21 epxression. Expression of GAPDH mRNA was used as control. Cell lysates were immunoblotted with Akt and actin antibodies. (B) Expression of miR-21 Sponge and Gag Akt for the results described in Fig. 6C. Expression of GFP mRNA and Akt protein was determined as described above.
(TIF)

Figure S14 (A) Expression of constitutively active Gag-Akt reversed mir-21 Sponge-induced inhibition of migration of Caki-2 renal cancer cells. Caki-2 cells were transfected either with miR-21 Sponge alone or along with Gag-Akt expression plasmid. Migration of the transfected cells were measured as described in the legend of Fig. 2C. (B) The absorbance of the stain of the migrated cells in panel A was determined. Mean ± SE of 3 measurements is shown. *p<0.001 vs control; **p<0.001 vs miR-21 Sponge. (C) Expression of miR-21 Sponge and Akt for the results described in panels A and B. Total RNAs and cell lysates were prepared from Caki-2 cells plated independently. GFP mRNA was detected as a surrogate for miR-21 Sponge expression. GAPDH was used as control. Cell lysates were immunoblotted with Akt and actin antibodies.
(TIF)

Figure S15 **Expression of miR-21 Sponge for the results presented in** **Fig. 7** **.** (A) ACHN cells were transfected with miR-21 Sponge or vector as indicated in the Figure 7A–7C. (B and C) ACHN cells were transfected either with miR-21 Sponge alone or along with CA Rheb expression plasmid as indicated in Fig. D and 7E, respectively. Total RNAs were used in RT-PCR for the detection of GFP mRNA, which serves as a surrogate for the expression of miR-21-neutralizing “Sponge” sequence. Detection of GAPDH mRNA was used as control.
(TIF)

Figure S16 **Expression of miR-21 Sponge and mTOR for the results described in** **Fig. 8A** **.** Total RNAs and cell lysates were used from miR-21 Sponge and constitutively active (CA) mTOR-transfected ACHN cells. GFP mRNA and GAPDH were detected. Cell lysates were immunoblotted with mTOR and actin antibodies.
(TIF)

Figure S17 (A) Expression of constitutively active mTOR reversed mir-21 Sponge-induced inhibition of DNA synthesis in Caki-2 renal cancer cells.^3^H-thymidine incorporation was used as a measure of DNA synthesis as described in the legend of Fig. 2A. Mean ± SE of 6 measurements is shown. *p<0.05 vs control; **p<0.001 vs miR-21 Sponge. (B) Expression of miR-21 Sponge and mTOR for the results described in panel A. Total RNAs and cell lysates were used from miR-21 Sponge and CA mTOR-transfected Caki-2 cells. GFP mRNA was used as a surrogate for miR-21 expression. Expression of GAPDH mRNA was used as control. Cell lysates were immunoblotted with mTOR and actin antibodies.
(TIF)

Figure S18 (A) Expression of miR-21 Sponge and mTOR for the results described in Fig. 8B. Total RNAs and cell lysates were used from miR-21 Sponge and constitutively active (CA) mTOR-transfected ACHN cells. GFP mRNA and GAPDH were detected. Cell lysates were immunoblotted with mTOR and actin antibodies. (B) Expression of miR-21 Sponge and mTOR for the results described in Figs. 8C and 8D. Total RNAs were used to detect GFP and GAPDH and cell lysates were used for immunoblotting with mTOR and actin antibodies.
(TIF)

Figure S19 (A) Expression of constitutively active mTOR reversed mir-21 Sponge-induced inhibition of migration of Caki-2 renal cancer cells. Caki-2 cells were transfected either with miR-21 Sponge alone or along with CA mTOR expression plasmid. Migration of the transfected cells were measured as described in the legend of Fig. 2C. (B) The absorbance of the stain of the migrated cells in panel A was determined. Mean ± SE of 3 measurements is shown. *p<0.001 vs control; **p<0.001 vs miR-21 Sponge. (C) Expression of miR-21 Sponge and mTOR for the results described in panels A and B. Total RNAs and cell lysates were prepared from Caki-2 cells plated independently. GFP mRNA was detected as a surrogate for miR-21 Sponge expression. GAPDH was used as control. Cell lysates were immunoblotted with mTOR and actin antibodies.
(TIF)

Figure S20 **Expression of miR-21 in VHL negative 786-O renal cell carcinoma cells.** Total RNAs from HK2 normal proximal tubular epithelial cells and 786-O renal carcinoma cells were used to detect mature miR-21 as described in the text. Expression of U6 RNA was used to normalize the data. Mean ± SE of 4 measurements is shown. *p<0.0001 vs HK2.
(TIF)

Figure S21 **Expression of miR-21 in BT-20 breast cancer cells.** Total RNAs from MCF-10A normal breast epithelial cells and BT-20 mammary carcinoma cells were used to detect mature miR-21 as described in the text. Expression of U6 RNA was used to normalize the data. Mean ± SE of 4 measurements is shown. *p  = 0.0006 vs MCF10A.
(TIF)

Figure S22 (A) Downregulation of PTEN reversed miR-21 Sponge-induced inhibition of BT-20 human breast cancer cell proliferation. BT-20 cells were transfected either with miR-21 Sponge alone or along with siRNAs against PTEN. The cells were trypsinized and counted using hemocytometer at indicated times. Mean ± SE of 3 measurements is shown. *p<0.01 vs vector; **p<0.01 vs miR-21 Sponge. (B) Expression of miR-21 Sponge and PTEN for the results described in panel A. Total RNAs and cell lysates were used from miR-21 Sponge and PTEN siRNA-transfected BT-20 cells. GFP mRNA and GAPDH were detected as described. Cell lysates were immunoblotted with PTEN and actin antibodies.
(TIF)

Figure S23 (A) Downregulation of PTEN reversed miR-21 Sponge-induced inhibition of migration of BT-20 human breast cancer cells. BT-20 cells were transfected either with miR-21 Sponge alone or along with siRNAs against PTEN. Migration of the transfected cells were measured as described in the legend of Fig. 2C. (B) The absorbance of the stain of the migrated cells in panel A was determined. Mean ± SE of 3 measurements is shown. *p<0.05 vs vector; **p<0.05 vs miR-21 Sponge. (C) Expression of miR-21 Sponge and PTEN for the results described in panels A and B. Total RNAs and cell lysates were prepared from BT-20 cells plated independently. GFP mRNA was detected as a surrogate for miR-21 Sponge expression. GAPDH was used as control. Cell lysates were immunoblotted with PTEN and actin antibodies.
(TIF)

Figure S24 (A) Expression of constitutively active Gag-Akt reversed miR-21 Sponge-induced inhibition of BT-20 human breast cancer cell proliferation. BT-20 cells were transfected either with miR-21 Sponge alone or along with Gag-Akt expression plasmid. The cells were trypsinized and counted using hemocytometer at indicated times. Mean ± SE of 3 measurements is shown. *p<0.05 vs vector; **p<0.01 vs miR-21 Sponge. (B) Expression of miR-21 Sponge and Akt for the results described in panel A. Total RNAs and cell lysates were used from miR-21 Sponge and Gag-Akt-transfected BT-20 cells. GFP mRNA and GAPDH were detected as described. Cell lysates were immunoblotted with Akt and actin antibodies.
(TIF)

Figure S25 (A) Expression of constitutively active Gag-Akt reversed miR-21 Sponge-induced inhibition of migration of BT-20 human breast cancer cells. BT-20 cells were transfected either with miR-21 Sponge alone or along with Gag-Akt expression plasmid. Migration of the transfected cells were measured as described in the legend of Fig. 2C. (B) The absorbance of the stain of the migrated cells in panel A was determined. Mean ± SE of 3 measurements is shown. *p<0.01 vs vector; **p<0.05 vs miR-21 Sponge. (C) Expression of miR-21 Sponge and Akt for the results described in panels A and B. Total RNAs and cell lysates were prepared from BT-20 cells plated independently. GFP mRNA was detected as a surrogate for miR-21 Sponge expression. GAPDH was used as control. Cell lysates were immunoblotted with Akt and actin antibodies.
(TIF)
