# Supplementary figures and images for: microRNA-21 Governs TORC1 Activation in Renal Cancer Cell Proliferation and Invasion
Source: PLoS One. 2012 Jun 4;7(6):e37366. doi: 10.1371/journal.pone.0037366 (PMC3368259; doi:10.1371/journal.pone.0037366)

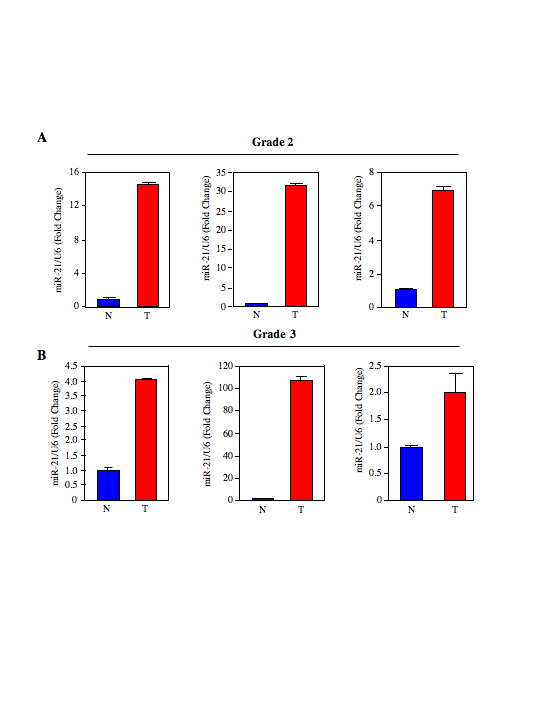

Supplement: Figure S1 — Expression of mature miR-21 in Grade 2 and Grade 3 clear cell renal carcinomas. Total RNAs from three renal tumor samples and from the normal portion of three kidneys were used for real time qRT-PCR to detect mature miR-21 as described in the text. The expression levels were normalized to U6. Each panel represents one subject. N, normal tissues; T, tumor tissues. (TIF) [file pone.0037366.s001.tif]

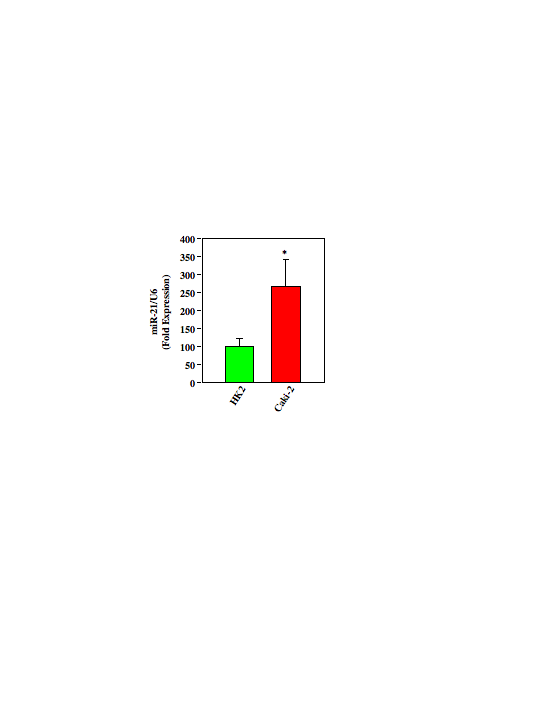

Supplement: Figure S2 — Expression of miR-21 in Caki-2 renal cancer cells. Total RNA from HK2 normal proximal tubular epithelial cells and Caki-2 cells was used for real time qRT-PCR to detect mature miR-21 as described in the text. The expression levels were normalized to U6. Mean ± SE of quadruplicate measurements is shown. *p = 0.03 vs HK2. (TIF) [file pone.0037366.s002.tif]

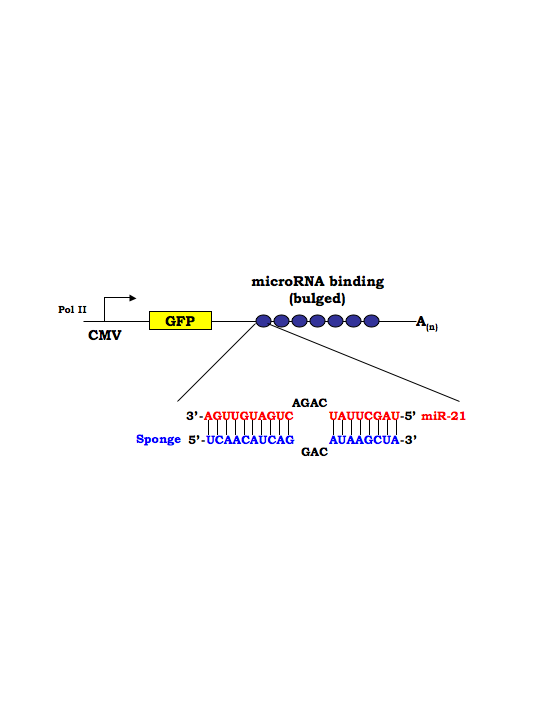

Supplement: Figure S3 — Structure of miR-21 Sponge expression of plasmid. Consecutive 7 copies of anti-miR-21 sequence with a bulge in each were introduced downstream of green fluorescence protein (GFP) cDNA. Sequence of the anti-miR-21 bulge is shown at the bottom. The GFP sequence is driven by RNA Pol II from the human cytomegalovirus (CMV) early promoter. (TIF) [file pone.0037366.s003.tif]

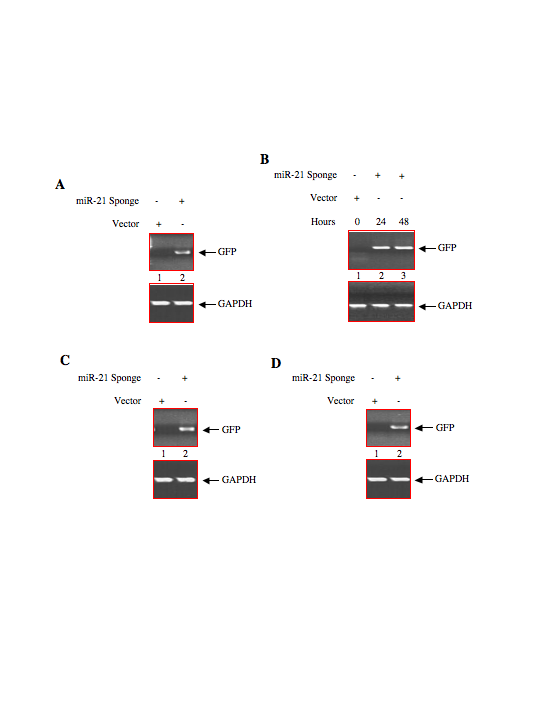

Supplement: Figure S4 — Expression of miR-21 Sponge for the results described in Fig. 2A – 2F. ACHN cells were transfected with miR-21 Sponge or vector plasmids as indicated in the Figure 2. Total RNAs were used in RT-PCR for the detection of GFP mRNA, which serves as the surrogate for the expression of miR-21 neutralizing “Sponge” sequence. Detection of GAPDH mRNA was used as control. Panels A and B represent data for Fig. 2A and 2B respectively. Panels C represents data for Fig. 2C and 2D. Panel D shows data for Fig. 2E and 2F, respectively. (TIF) [file pone.0037366.s004.tif]

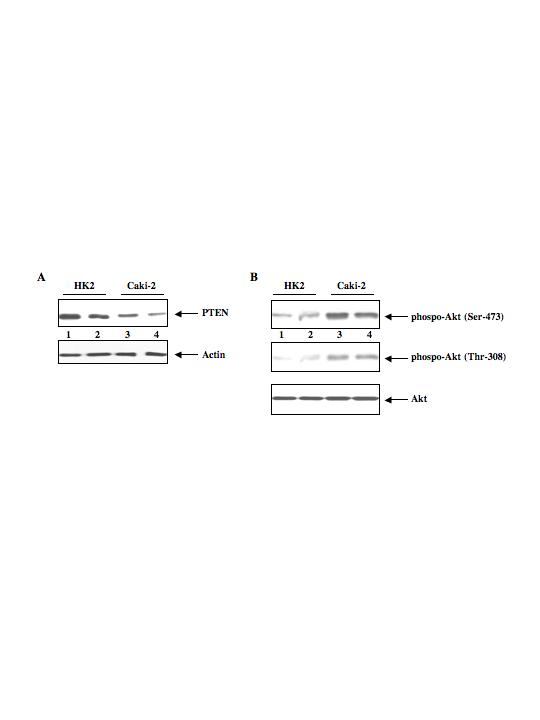

Supplement: Figure S5 — Expression of PTEN and activation of Akt in Caki-2 renal cancer cells. (A) Lysates of HK2 proximal tubular epithelial cells and Caki-2 cells were immunoblotted with PTEN and actin antibodies. (B) The same lyssates were immunoblotted with phospho-Akt (Ser-473 and Thr-308) and Akt antibodies as indicated. (TIF) [file pone.0037366.s005.tif]

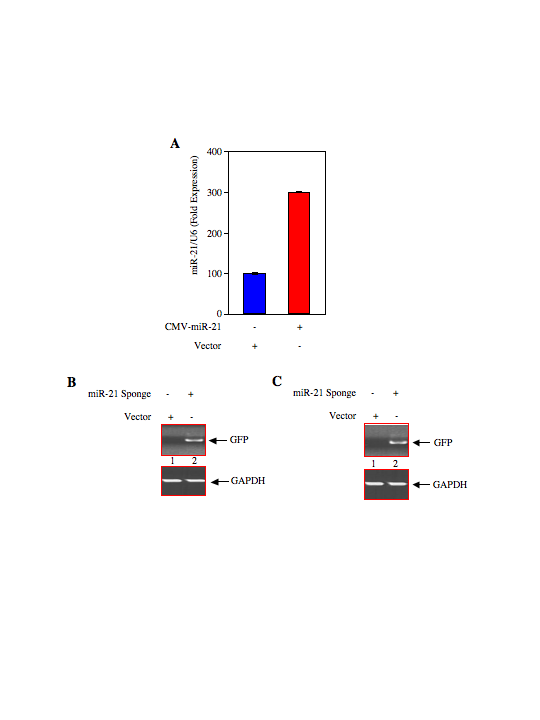

Supplement: Figure S6 — (A) Expression of mature miR-21 for the results described in Fig. 4A in the text. ACHN cells were transfected with CMV-miR-21 or Vector. The total RNAs were used to detect mature miR-21. The level of miR-21 was corrected for U6 RNA expression. (B) Expression of miR-21 Sponge for the results presented in Fig. 4B. (C) Expression of miR-21 Sponge for the results presented in Figs. 4C and 4D. Total RNAs were used to detect GFP mRNA and GAPDH as described in the Fig. S4. (TIF) [file pone.0037366.s006.tif]

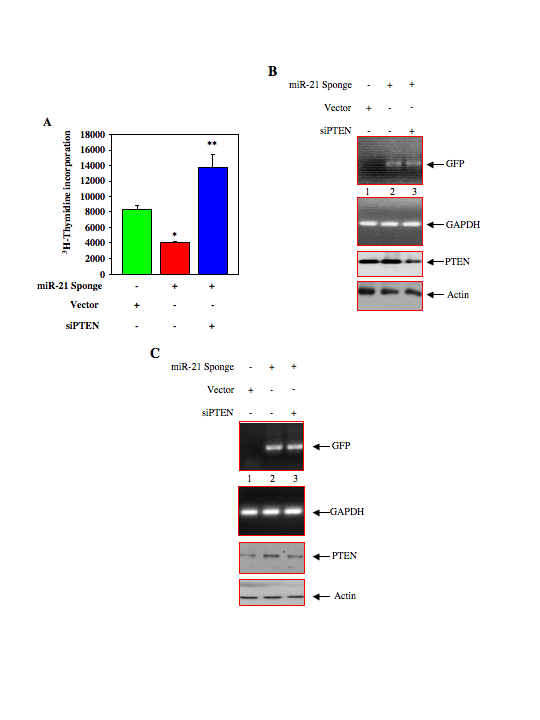

Supplement: Figure S7 — (A) Downregulation of PTEN reversed mir-21 Sponge-induced inhibition of DNA synthesis in Caki-2 renal cancer cells.3H-thymidine incorporation was used as a measure of DNA synthesis as described in the legend of Fig. 2A. Mean ± SE of 6 measurements is shown. *p<0.05 vs control; **p<0.01 vs miR-21 Sponge. (B) Expression of miR-21 Sponge and PTEN for the results described in panel A. Total RNAs and cell lysates were used from miR-21 Sponge and PTEN siRNA-transfected Caki-2 cells. GFP mRNA and GAPDH were detected as described in the Supplemental Fig. S4. Cell lysates were immunoblotted with PTEN and actin antibodies. (C) Expression of miR-21 Sponge and PTEN for the results described in Fig. 5A. Total RNAs and cell lysates were used from miR-21 Sponge and PTEN siRNA-transfected ACHN cells. GFP and GAPDH mRNAs were detected as described in the Supplemental Fig. S4. Cell lysates were immunoblotted with PTEN and actin antibodies. (TIF) [file pone.0037366.s007.tif]

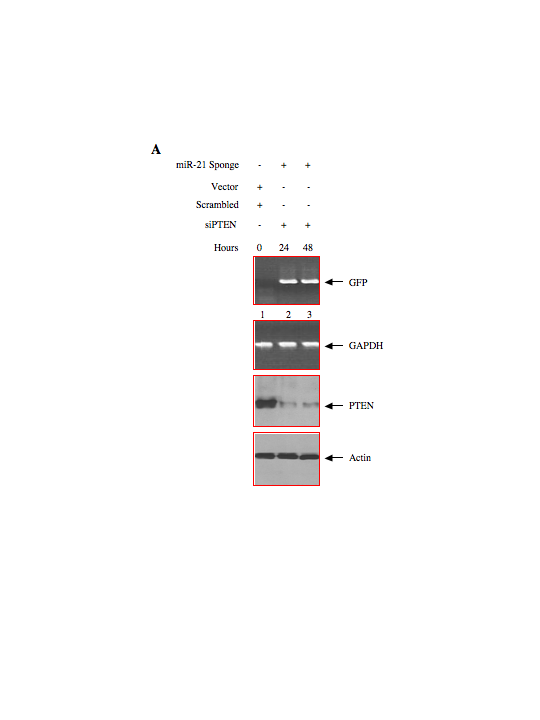

Supplement: Figure S8 — Expression of miR-21 Sponge and PTEN for the results described in Fig. 5B . Expression of GFP and GAPDH mRNAs and PTEN and actin proteins were determined as described in the legend of Fig. S7C. Expression of miR-21 Sponge for the results described in Figs. 5C and 5D. Total RNAs were used to detect GFP and GAPDH and cell lysates were used for immunoblotting with PTEN and actin antibodies. (TIF) [file pone.0037366.s008.tif]

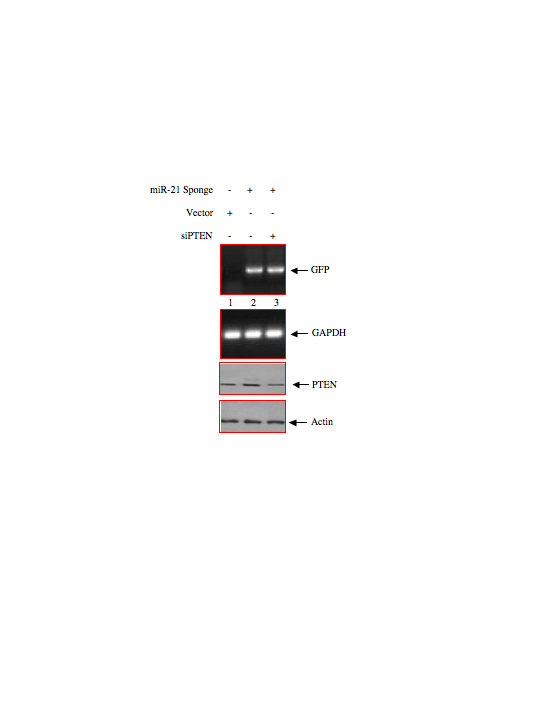

Supplement: Figure S9 — Expression of miR-21 Sponge for the results described in Figs. 5C and 5D . Total RNAs were used to detect GFP and GAPDH and cell lysates were used for immunoblotting with PTEN and actin antibodies. (TIF) [file pone.0037366.s009.tif]

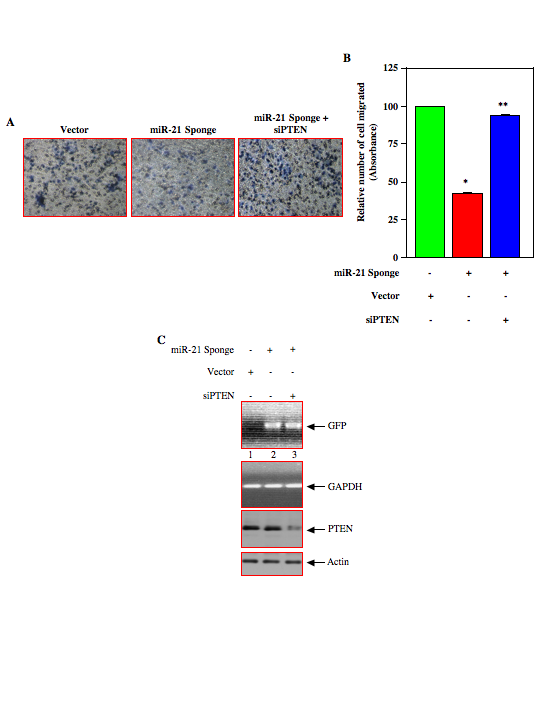

Supplement: Figure S10 — (A) Downregulation of PTEN reversed mir-21 Sponge-induced inhibition of migration of Caki-2 renal cancer cells. Caki-2 cells were transfected either with miR-21 Sponge alone or along with siRNAs against PTEN. Migration of the transfected cells were measured as described in the legend of Fig. 2C. (B) The absorbance of the stain of the migrated cells in panel A was determined. Mean ± SE of 3 measurements is shown. *p<0.001 vs control; **p<0.001 vs miR-21 Sponge. (C) Expression of miR-21 Sponge and PTEN for the results described in panels A and B. Total RNAs and cell lysates were prepared from Caki-2 cells plated independently. GFP mRNA was detected as a surrogate for miR-21 Sponge expression. GAPDH was used as control. Cell lysates were immunoblotted with PTEN and actin antibodies. (TIF) [file pone.0037366.s010.tif]

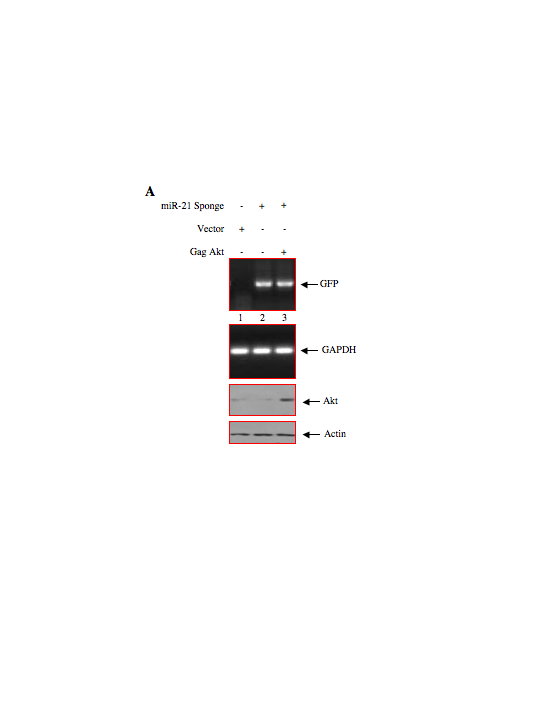

Supplement: Figure S11 — Expression of miR-21 Sponge and Akt for the results described in Fig. 6A . Total RNAs and cell lysates were used from miR-21 Sponge and Gag Akt-transfected ACHN cells. GFP mRNA and GAPDH were detected. (TIF) [file pone.0037366.s011.tif]

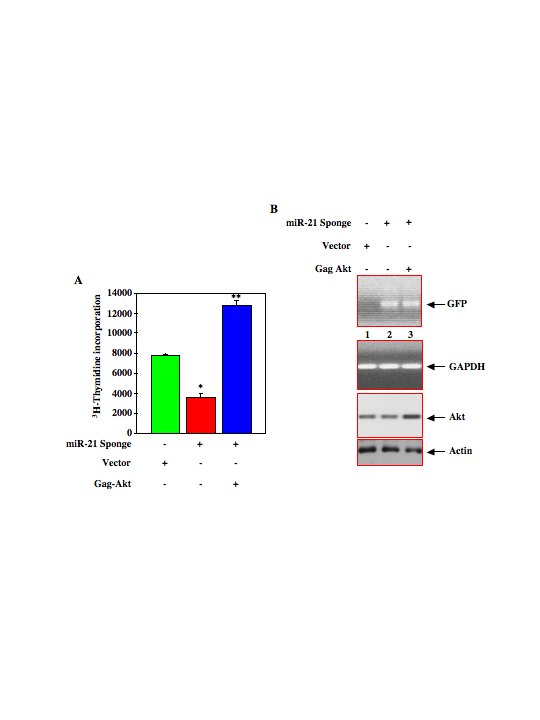

Supplement: Figure S12 — (A) Expression of constitutively active Gag-Akt reversed mir-21 Sponge-induced inhibition of DNA synthesis in Caki-2 renal cancer cells.3H-thymidine incorporation was used as a measure of DNA synthesis as described in the legend of Fig. 2A. Mean ± SE of 6 measurements is shown. *p<0.01 vs control; **p<0.001 vs miR-21 Sponge. (B) Expression of miR-21 Sponge and Akt for the results described in panel A. Total RNAs and cell lysates were used from miR-21 Sponge and Gag-Akt-transfected Caki-2 cells. GFP mRNA was used as a surrogate for miR-21 epxression. Expression of GAPDH mRNA was used as control. Cell lysates were immunoblotted with Akt and actin antibodies. (TIF) [file pone.0037366.s012.tif]

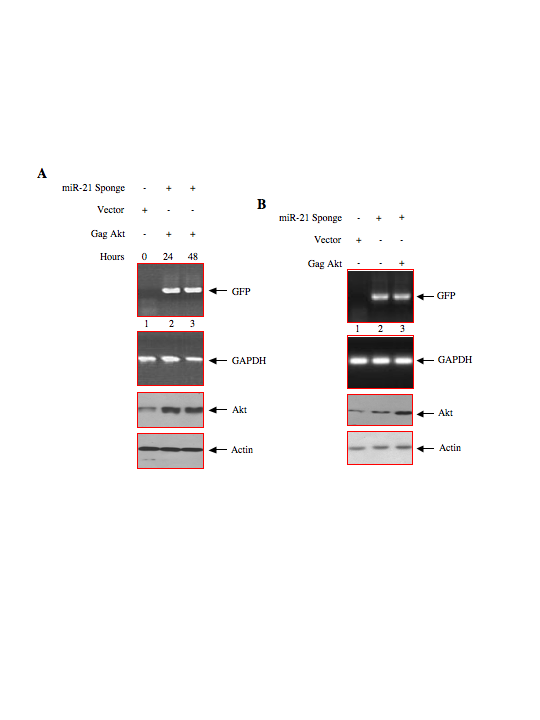

Supplement: Figure S13 — (A) Expression of miR-21 Sponge and Gag Akt for the results described in Fig. 6B. Total RNAs and cell lysates were used from miR-21 Sponge and Gag-Akt-transfectedACHNcells. GFP mRNA was used as a surrogate for miR-21 epxression. Expression of GAPDH mRNA was used as control. Cell lysates were immunoblotted with Akt and actin antibodies. (B) Expression of miR-21 Sponge and Gag Akt for the results described in Fig. 6C. Expression of GFP mRNA and Akt protein was determined as described above. (TIF) [file pone.0037366.s013.tif]

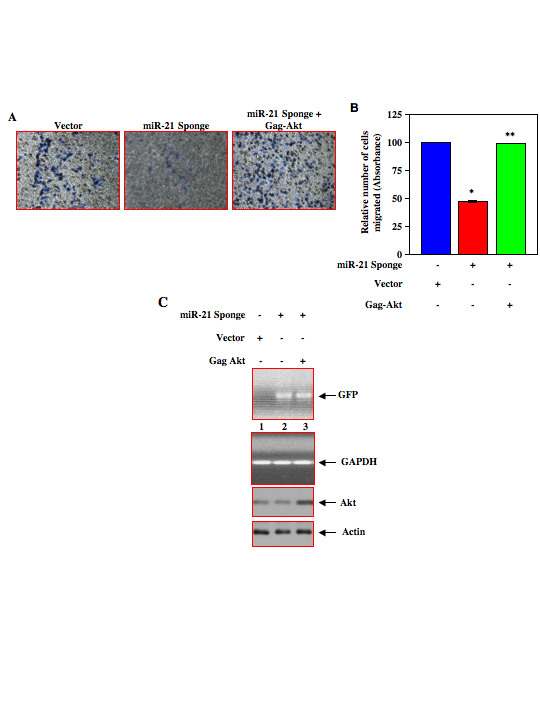

Supplement: Figure S14 — (A) Expression of constitutively active Gag-Akt reversed mir-21 Sponge-induced inhibition of migration of Caki-2 renal cancer cells. Caki-2 cells were transfected either with miR-21 Sponge alone or along with Gag-Akt expression plasmid. Migration of the transfected cells were measured as described in the legend of Fig. 2C. (B) The absorbance of the stain of the migrated cells in panel A was determined. Mean ± SE of 3 measurements is shown. *p<0.001 vs control; **p<0.001 vs miR-21 Sponge. (C) Expression of miR-21 Sponge and Akt for the results described in panels A and B. Total RNAs and cell lysates were prepared from Caki-2 cells plated independently. GFP mRNA was detected as a surrogate for miR-21 Sponge expression. GAPDH was used as control. Cell lysates were immunoblotted with Akt and actin antibodies. (TIF) [file pone.0037366.s014.tif]

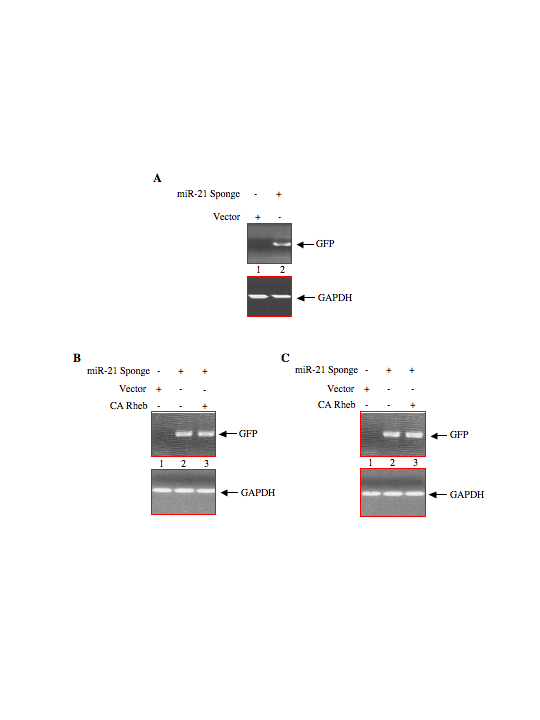

Supplement: Figure S15 — Expression of miR-21 Sponge for the results presented in Fig. 7 . (A) ACHN cells were transfected with miR-21 Sponge or vector as indicated in the Figure 7A–7C. (B and C) ACHN cells were transfected either with miR-21 Sponge alone or along with CA Rheb expression plasmid as indicated in Fig. D and 7E, respectively. Total RNAs were used in RT-PCR for the detection of GFP mRNA, which serves as a surrogate for the expression of miR-21-neutralizing “Sponge” sequence. Detection of GAPDH mRNA was used as control. (TIF) [file pone.0037366.s015.tif]

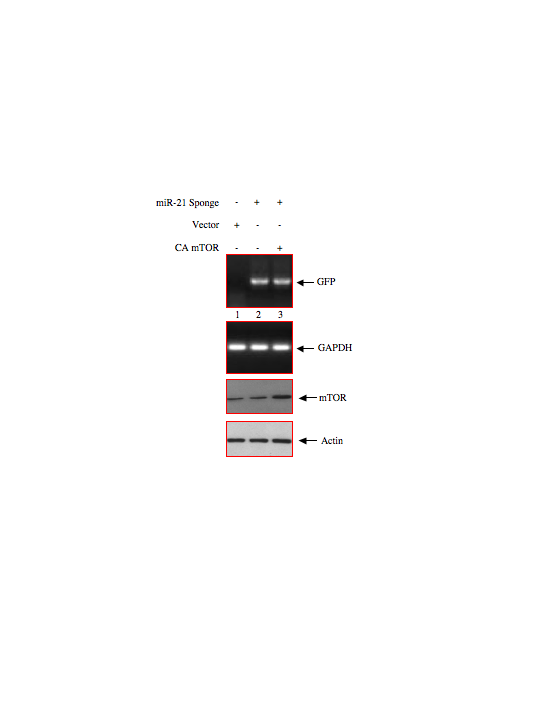

Supplement: Figure S16 — Expression of miR-21 Sponge and mTOR for the results described in Fig. 8A . Total RNAs and cell lysates were used from miR-21 Sponge and constitutively active (CA) mTOR-transfected ACHN cells. GFP mRNA and GAPDH were detected. Cell lysates were immunoblotted with mTOR and actin antibodies. (TIF) [file pone.0037366.s016.tif]

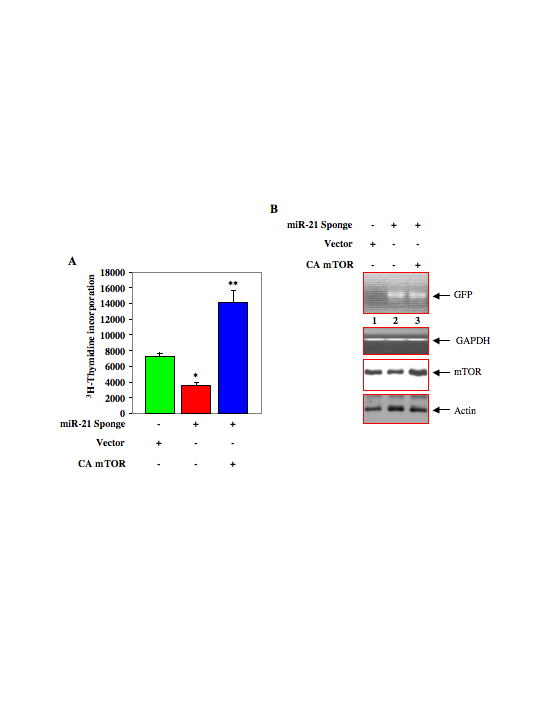

Supplement: Figure S17 — (A) Expression of constitutively active mTOR reversed mir-21 Sponge-induced inhibition of DNA synthesis in Caki-2 renal cancer cells.3H-thymidine incorporation was used as a measure of DNA synthesis as described in the legend of Fig. 2A. Mean ± SE of 6 measurements is shown. *p<0.05 vs control; **p<0.001 vs miR-21 Sponge. (B) Expression of miR-21 Sponge and mTOR for the results described in panel A. Total RNAs and cell lysates were used from miR-21 Sponge and CA mTOR-transfected Caki-2 cells. GFP mRNA was used as a surrogate for miR-21 expression. Expression of GAPDH mRNA was used as control. Cell lysates were immunoblotted with mTOR and actin antibodies. (TIF) [file pone.0037366.s017.tif]

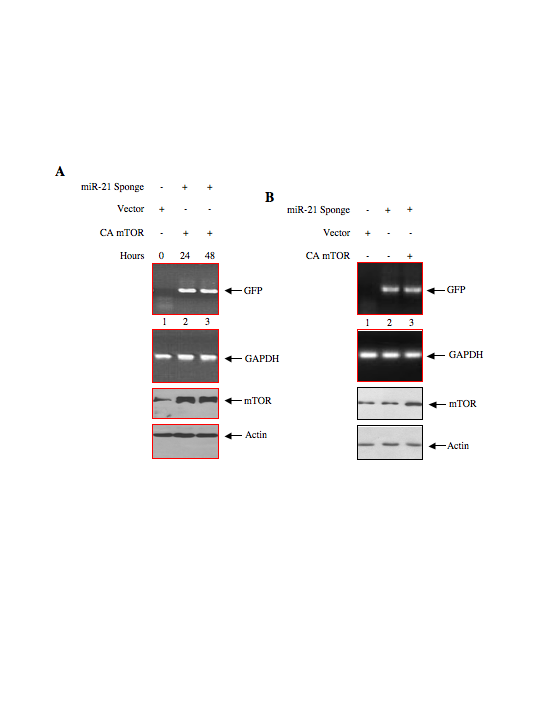

Supplement: Figure S18 — (A) Expression of miR-21 Sponge and mTOR for the results described in Fig. 8B. Total RNAs and cell lysates were used from miR-21 Sponge and constitutively active (CA) mTOR-transfected ACHN cells. GFP mRNA and GAPDH were detected. Cell lysates were immunoblotted with mTOR and actin antibodies. (B) Expression of miR-21 Sponge and mTOR for the results described in Figs. 8C and 8D. Total RNAs were used to detect GFP and GAPDH and cell lysates were used for immunoblotting with mTOR and actin antibodies. (TIF) [file pone.0037366.s018.tif]

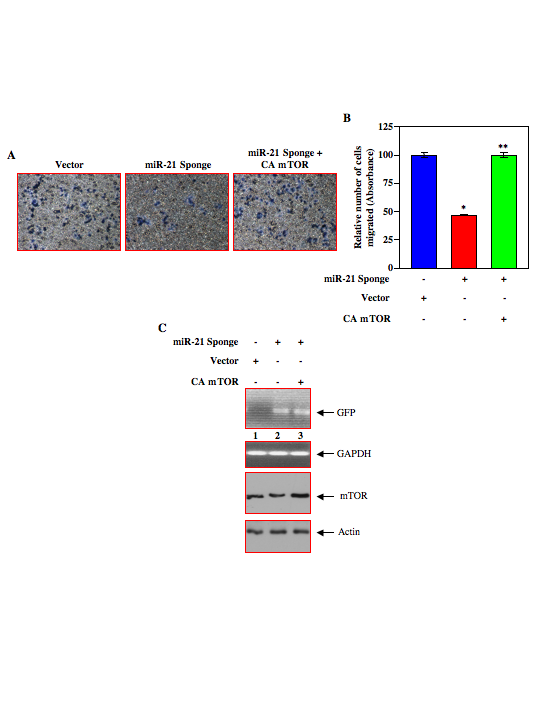

Supplement: Figure S19 — (A) Expression of constitutively active mTOR reversed mir-21 Sponge-induced inhibition of migration of Caki-2 renal cancer cells. Caki-2 cells were transfected either with miR-21 Sponge alone or along with CA mTOR expression plasmid. Migration of the transfected cells were measured as described in the legend of Fig. 2C. (B) The absorbance of the stain of the migrated cells in panel A was determined. Mean ± SE of 3 measurements is shown. *p<0.001 vs control; **p<0.001 vs miR-21 Sponge. (C) Expression of miR-21 Sponge and mTOR for the results described in panels A and B. Total RNAs and cell lysates were prepared from Caki-2 cells plated independently. GFP mRNA was detected as a surrogate for miR-21 Sponge expression. GAPDH was used as control. Cell lysates were immunoblotted with mTOR and actin antibodies. (TIF) [file pone.0037366.s019.tif]

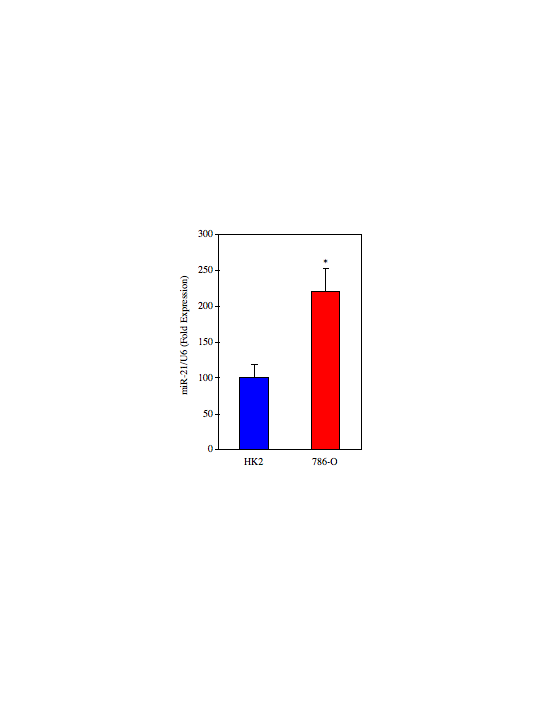

Supplement: Figure S20 — Expression of miR-21 in VHL negative 786-O renal cell carcinoma cells. Total RNAs from HK2 normal proximal tubular epithelial cells and 786-O renal carcinoma cells were used to detect mature miR-21 as described in the text. Expression of U6 RNA was used to normalize the data. Mean ± SE of 4 measurements is shown. *p<0.0001 vs HK2. (TIF) [file pone.0037366.s020.tif]

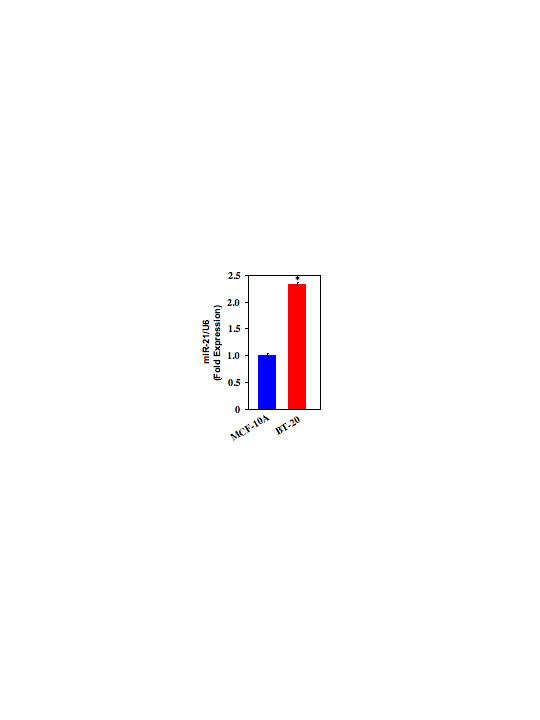

Supplement: Figure S21 — Expression of miR-21 in BT-20 breast cancer cells. Total RNAs from MCF-10A normal breast epithelial cells and BT-20 mammary carcinoma cells were used to detect mature miR-21 as described in the text. Expression of U6 RNA was used to normalize the data. Mean ± SE of 4 measurements is shown. *p = 0.0006 vs MCF10A. (TIF) [file pone.0037366.s021.tif]

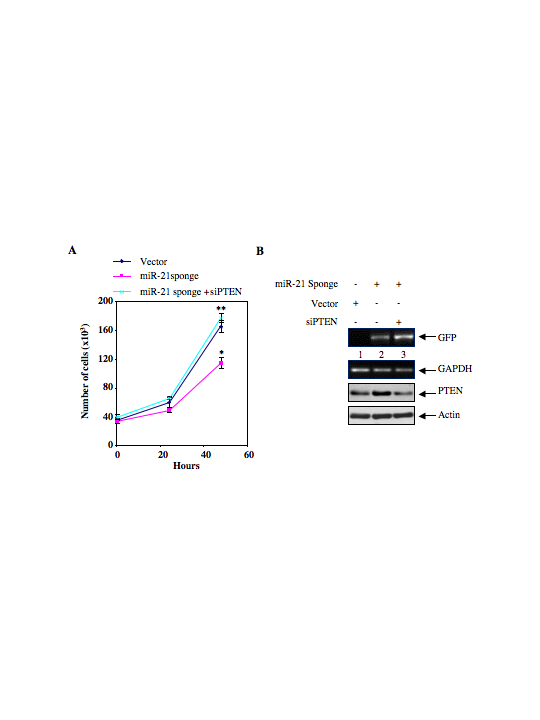

Supplement: Figure S22 — (A) Downregulation of PTEN reversed miR-21 Sponge-induced inhibition of BT-20 human breast cancer cell proliferation. BT-20 cells were transfected either with miR-21 Sponge alone or along with siRNAs against PTEN. The cells were trypsinized and counted using hemocytometer at indicated times. Mean ± SE of 3 measurements is shown. *p<0.01 vs vector; **p<0.01 vs miR-21 Sponge. (B) Expression of miR-21 Sponge and PTEN for the results described in panel A. Total RNAs and cell lysates were used from miR-21 Sponge and PTEN siRNA-transfected BT-20 cells. GFP mRNA and GAPDH were detected as described. Cell lysates were immunoblotted with PTEN and actin antibodies. (TIF) [file pone.0037366.s022.tif]

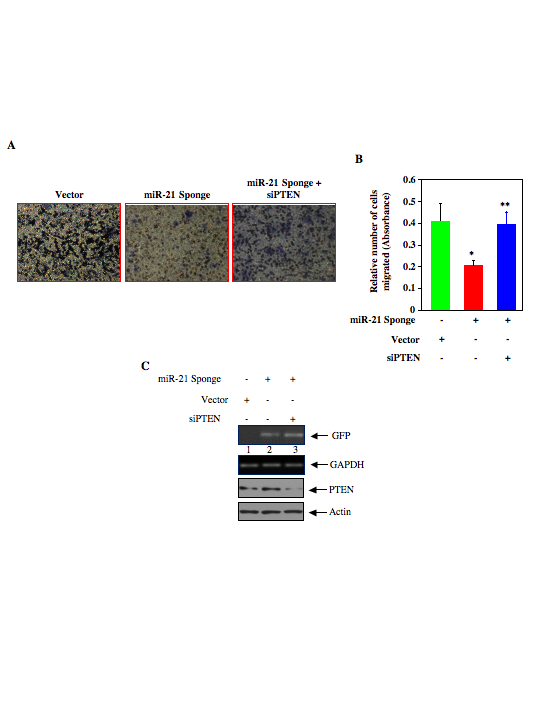

Supplement: Figure S23 — (A) Downregulation of PTEN reversed miR-21 Sponge-induced inhibition of migration of BT-20 human breast cancer cells. BT-20 cells were transfected either with miR-21 Sponge alone or along with siRNAs against PTEN. Migration of the transfected cells were measured as described in the legend of Fig. 2C. (B) The absorbance of the stain of the migrated cells in panel A was determined. Mean ± SE of 3 measurements is shown. *p<0.05 vs vector; **p<0.05 vs miR-21 Sponge. (C) Expression of miR-21 Sponge and PTEN for the results described in panels A and B. Total RNAs and cell lysates were prepared from BT-20 cells plated independently. GFP mRNA was detected as a surrogate for miR-21 Sponge expression. GAPDH was used as control. Cell lysates were immunoblotted with PTEN and actin antibodies. (TIF) [file pone.0037366.s023.tif]

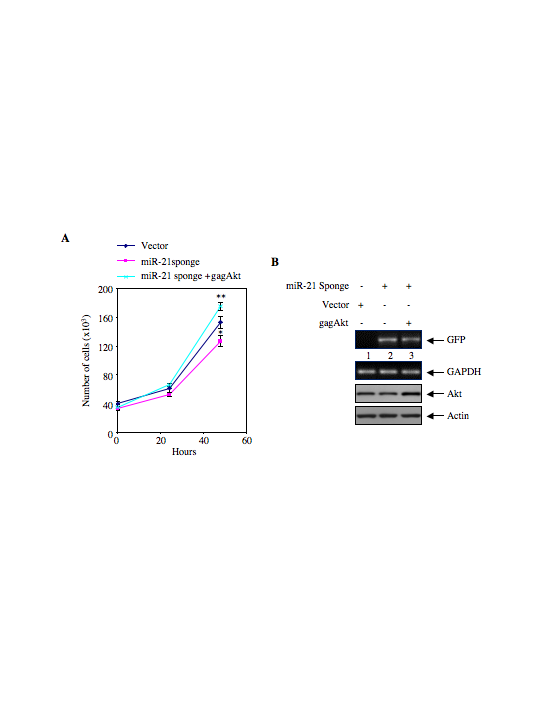

Supplement: Figure S24 — (A) Expression of constitutively active Gag-Akt reversed miR-21 Sponge-induced inhibition of BT-20 human breast cancer cell proliferation. BT-20 cells were transfected either with miR-21 Sponge alone or along with Gag-Akt expression plasmid. The cells were trypsinized and counted using hemocytometer at indicated times. Mean ± SE of 3 measurements is shown. *p<0.05 vs vector; **p<0.01 vs miR-21 Sponge. (B) Expression of miR-21 Sponge and Akt for the results described in panel A. Total RNAs and cell lysates were used from miR-21 Sponge and Gag-Akt-transfected BT-20 cells. GFP mRNA and GAPDH were detected as described. Cell lysates were immunoblotted with Akt and actin antibodies. (TIF) [file pone.0037366.s024.tif]

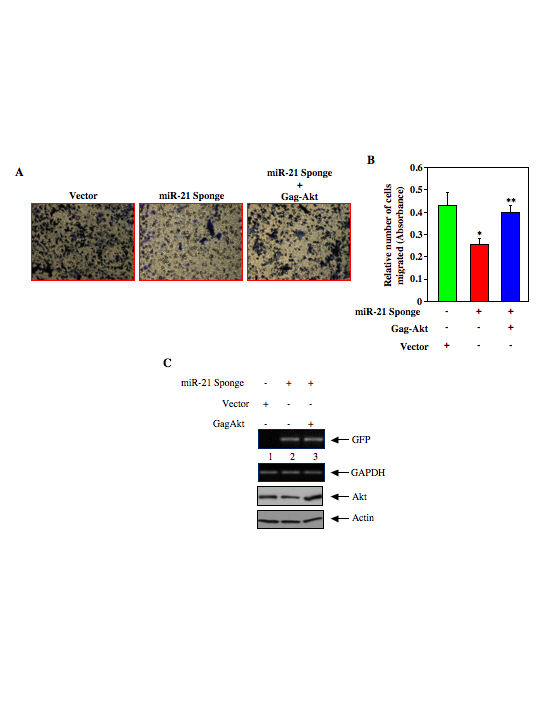

Supplement: Figure S25 — (A) Expression of constitutively active Gag-Akt reversed miR-21 Sponge-induced inhibition of migration of BT-20 human breast cancer cells. BT-20 cells were transfected either with miR-21 Sponge alone or along with Gag-Akt expression plasmid. Migration of the transfected cells were measured as described in the legend of Fig. 2C. (B) The absorbance of the stain of the migrated cells in panel A was determined. Mean ± SE of 3 measurements is shown. *p<0.01 vs vector; **p<0.05 vs miR-21 Sponge. (C) Expression of miR-21 Sponge and Akt for the results described in panels A and B. Total RNAs and cell lysates were prepared from BT-20 cells plated independently. GFP mRNA was detected as a surrogate for miR-21 Sponge expression. GAPDH was used as control. Cell lysates were immunoblotted with Akt and actin antibodies. (TIF) [file pone.0037366.s025.tif]
